# Cell-free DNA Fragmentomics Assay to Discriminate the Malignancy of Breast Nodules and Evaluate Treatment Response

**DOI:** 10.1093/gpbjnl/qzaf028

**Published:** 2025-04-04

**Authors:** Jiaqi Liu, Yalun Li, Wanxiangfu Tang, Tianyi Qian, Lijun Dai, Ziqi Jia, Heng Cao, Chenghao Li, Yuchen Liu, Yansong Huang, Jiang Wu, Dongxu Ma, Guangdong Qiao, Hua Bao, Shuang Chang, Dongqin Zhu, Shanshan Yang, Xuxiaochen Wu, Xue Wu, Hengyi Xu, Hongyan Chen, Yang Shao, Xiang Wang, Zhihua Liu, Jianzhong Su

**Affiliations:** State Key Laboratory of Molecular Oncology, National Cancer Center/National Clinical Research Center for Cancer/Cancer Hospital, Chinese Academy of Medical Sciences and Peking Union Medical College, Beijing 100021, China; Oujiang Laboratory (Zhejiang Lab for Regenerative Medicine, Vision and Brain Health), Eye Hospital, Wenzhou Medical University, Wenzhou 325027, China; Department of Breast Surgical Oncology, National Cancer Center/National Clinical Research Center for Cancer/Cancer Hospital, Chinese Academy of Medical Sciences and Peking Union Medical College, Beijing 100021, China; Department of Breast Surgery, Yantai Yuhuangding Hospital, The Affiliated Hospital of Qingdao University, Yantai 264000, China; Nanjing Geneseeq Technology Inc., Nanjing 210061, China; Department of Breast Surgery, The Second Affiliated Hospital, Zhejiang University School of Medicine, Hangzhou 310009, China; Oujiang Laboratory (Zhejiang Lab for Regenerative Medicine, Vision and Brain Health), Eye Hospital, Wenzhou Medical University, Wenzhou 325027, China; Department of Breast Surgical Oncology, National Cancer Center/National Clinical Research Center for Cancer/Cancer Hospital, Chinese Academy of Medical Sciences and Peking Union Medical College, Beijing 100021, China; Department of Breast Surgical Oncology, National Cancer Center/National Clinical Research Center for Cancer/Cancer Hospital, Chinese Academy of Medical Sciences and Peking Union Medical College, Beijing 100021, China; Oujiang Laboratory (Zhejiang Lab for Regenerative Medicine, Vision and Brain Health), Eye Hospital, Wenzhou Medical University, Wenzhou 325027, China; Department of Breast Surgical Oncology, National Cancer Center/National Clinical Research Center for Cancer/Cancer Hospital, Chinese Academy of Medical Sciences and Peking Union Medical College, Beijing 100021, China; School of Clinical Medicine, Chinese Academy of Medical Sciences and Peking Union Medical College, Beijing 100005, China; Department of Breast Surgical Oncology, National Cancer Center/National Clinical Research Center for Cancer/Cancer Hospital, Chinese Academy of Medical Sciences and Peking Union Medical College, Beijing 100021, China; School of Clinical Medicine, Chinese Academy of Medical Sciences and Peking Union Medical College, Beijing 100005, China; Department of Breast Surgical Oncology, National Cancer Center/National Clinical Research Center for Cancer/Cancer Hospital, Chinese Academy of Medical Sciences and Peking Union Medical College, Beijing 100021, China; Department of Breast Surgical Oncology, National Cancer Center/National Clinical Research Center for Cancer/Cancer Hospital, Chinese Academy of Medical Sciences and Peking Union Medical College, Beijing 100021, China; Department of Breast Surgery, Yantai Yuhuangding Hospital, The Affiliated Hospital of Qingdao University, Yantai 264000, China; Nanjing Geneseeq Technology Inc., Nanjing 210061, China; Nanjing Geneseeq Technology Inc., Nanjing 210061, China; Nanjing Geneseeq Technology Inc., Nanjing 210061, China; Nanjing Geneseeq Technology Inc., Nanjing 210061, China; Nanjing Geneseeq Technology Inc., Nanjing 210061, China; Nanjing Geneseeq Technology Inc., Nanjing 210061, China; State Key Laboratory of Molecular Oncology, National Cancer Center/National Clinical Research Center for Cancer/Cancer Hospital, Chinese Academy of Medical Sciences and Peking Union Medical College, Beijing 100021, China; School of Clinical Medicine, Chinese Academy of Medical Sciences and Peking Union Medical College, Beijing 100005, China; State Key Laboratory of Molecular Oncology, National Cancer Center/National Clinical Research Center for Cancer/Cancer Hospital, Chinese Academy of Medical Sciences and Peking Union Medical College, Beijing 100021, China; Nanjing Geneseeq Technology Inc., Nanjing 210061, China; Department of Breast Surgical Oncology, National Cancer Center/National Clinical Research Center for Cancer/Cancer Hospital, Chinese Academy of Medical Sciences and Peking Union Medical College, Beijing 100021, China; State Key Laboratory of Molecular Oncology, National Cancer Center/National Clinical Research Center for Cancer/Cancer Hospital, Chinese Academy of Medical Sciences and Peking Union Medical College, Beijing 100021, China; Oujiang Laboratory (Zhejiang Lab for Regenerative Medicine, Vision and Brain Health), Eye Hospital, Wenzhou Medical University, Wenzhou 325027, China

**Keywords:** Cell-free DNA fragmentomics, Cell-free DNA methylation, Breast cancer, Whole-genome sequencing, Neoadjuvant chemotherapy

## Abstract

The fragmentomics-based cell-free DNA (cfDNA) assays have recently illustrated prominent abilities to identify various cancers from non-conditional healthy controls, while their accuracy for identifying early-stage cancers from benign lesions with inconclusive imaging results remains uncertain. Especially for breast cancer, current imaging-based screening methods suffer from high false positive rates for women with breast nodules, leading to unnecessary biopsies, which add to discomfort and healthcare burden. Here, we enrolled 613 female participants in this multi-center study and demonstrated that cfDNA fragmentomics (cfFrag) is a robust non-invasive biomarker for breast cancer using whole-genome sequencing. Among the multimodal cfFrag profiles, the fragment size ratio (FSR), fragment size distribution (FSD), and copy number variation (CNV) show more distinguishing ability than Griffin, motif breakpoint (MBP), and neomer. The cfFrag model using the optimal three fragmentomics features discriminated early-stage breast cancer from benign nodules, even at a low sequencing depth (3×). Notably, it demonstrated a specificity of 94.1% in asymptomatic healthy women at a 90% sensitivity for breast cancer. Moreover, we comprehensively showcased the clinical utility of the cfFrag model in predicting patient responses to neoadjuvant chemotherapy (NAC) and its enhanced performance when combined with multimodal features, including radiological results [area under the curve (AUC) = 0.93–0.94] and cfDNA methylation features (AUC = 0.96).

## Graphical Abstract

**Figure:**
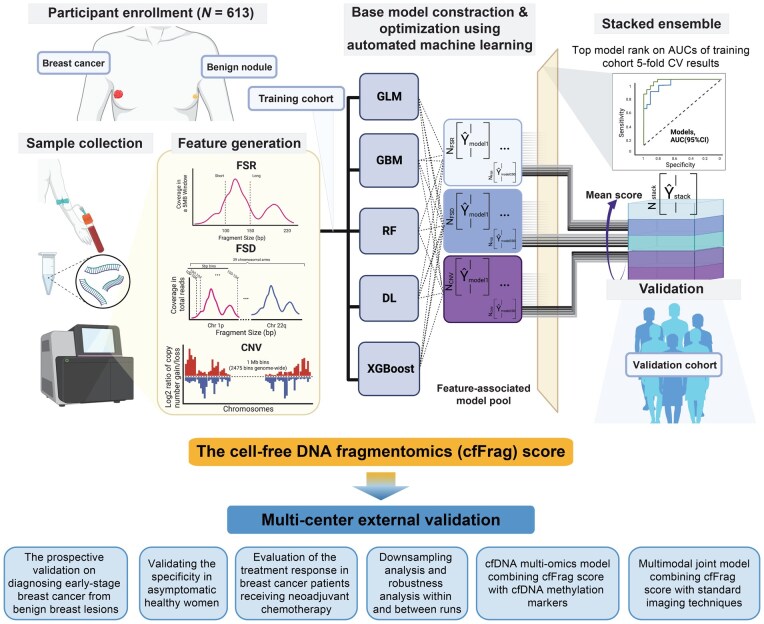

## Teaser

**Highlights**
 

• This is the first study to systematically evaluate the performance of cfDNA fragmentomics in distinguishing early-stage cancers from benign conditions with similar symptoms or inconclusive imaging results.
• The low-depth cfDNA fragmentomics profiling combined with automated machine learning demonstrates excellent performance in the early detection of breast cancer and prediction of neoadjuvant chemotherapy response in a multi-center prospective study.
• The cfFrag model maintains its predictive power even at a low sequencing depth (3×).
• The combination of non-invasive cfDNA fragmentomics features with standard diagnostic imaging or cfDNA methylomics features would further improve early breast cancer detection accuracy.

## Introduction

Breast cancer is one of the most common types of cancer worldwide and accounts for the highest number of cancer-related deaths among females [1]. Early detection of breast cancer is crucial for improving patients’ outcomes and survival [2]. However, current imaging-based screening methodologies, including mammography and ultrasonography, suffer from high false positive rates, leading to many unnecessary biopsies, adding to patient discomfort [3]. Meanwhile, tumor biomarkers such as CA15-3 lack sensitivity for early-stage breast cancer [4]. Thus, liquid biopsies are needed as a non-invasive alternative or adjunct to select the high breast cancer risk women for tumor biopsies [5].

Mutation-based circulating tumor DNA (ctDNA) detection has become the companion diagnostic by identifying actionable targets and alterations mediating resistance (*e.g.*, *ESR1* and *PIK3CA* mutations in breast cancer) [6–8]. However, ctDNA typically lacks mutations, especially in early-stage disease, which limits its application in these contexts and reduces its ability to anticipate the diagnosis of localized cancer [9]. Besides, lacking common mutations in breast cancer limits the detection sensitivity in the patient-naïve approach [10]. Epigenetic analysis approaches offer potential solutions to fully exploit liquid biopsy in various settings [11,12]. We previously conducted a whole-genome DNA methylation analysis on cell-free DNA (cfDNA) and identified ten optimal DNA methylation markers associated with breast cancer, which could enhance early detection [4]. However, current bisulfite-based methylation sequencing is prone to cfDNA damage, resulting in high cfDNA amount, depth dependencies, and increased cost.

Fragmentomics-based cfDNA assays have recently illustrated prominent abilities to identify various cancer types from paired non-conditional healthy controls using whole-genome sequencing (WGS) [13–17] and targeted cfDNA panels [18]. Similar to most cancer types, benign tumors also release ctDNA with unique features [19]. However, the accuracies of the cfDNA fragmentomics (cfFrag) profile for identifying early-stage cancers from benign lesions with similar symptoms or inconclusive imaging results and predicting the therapeutic response remain largely unclear.

Herein, we developed a non-invasive liquid biopsy assay for early-stage breast cancer diagnosis that analyzes cfFrag through low-depth WGS and machine learning. To reveal its clinical utilities, we comprehensively evaluated the performance of this cfFrag assay in diagnosing early-stage breast cancer from benign breast nodules, predicting patient responses to neoadjuvant chemotherapy (NAC), and combining with multimodal features, including standard imaging techniques and cfDNA methylation markers. This approach is particularly beneficial for female patients who have undergone unnecessary biopsies due to false positives from imaging tests on benign breast nodules. Additionally, it can offer valuable insights into neoadjuvant treatment planning for breast cancer patients. Combining a cfFrag assay with standard imaging techniques enhances the early detection rate of breast cancer, potentially improving breast cancer survival rates.

## Results

### Patient characteristics in two independent cohorts

**Figure 1:**
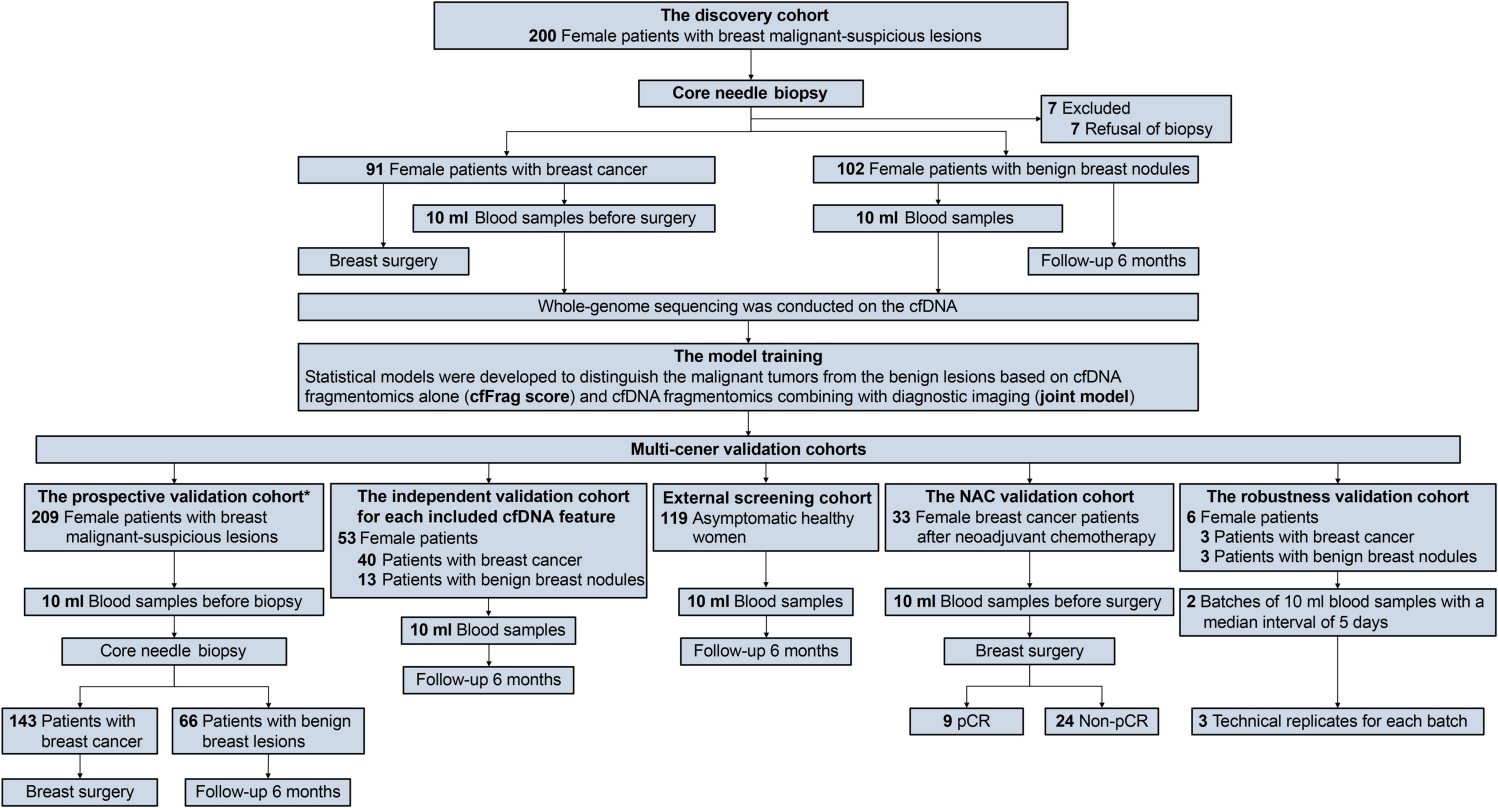
Patient enrollment In this multi-center study, we recruited 200 consecutive female patients with malignant-suspicious breast imaging results from the Yantai cohort as the training set. As a result, 91 patients with breast cancer and 102 women with benign breast nodules were enrolled, and 7 patients who refused to biopsy were excluded. The external validation cohorts were composed of three independent validation cohorts in Beijing, namely the prospective validation cohort (*N* = 209), the NAC validation cohort (*N* = 33), and the robustness analysis cohort (*N* = 6), a screening cohort of healthy women in Nanjing (*N* = 119), and an independent validation cohort for each included cDNA feature in Hangzhou (*N* = 53). *, the prospective validation cohort included 39 participants (14 with breast cancer and 25 with benign nodules) enrolled in a methylation-based early detection analysis for breast cancer through whole-genome bisulfite sequencing. cfDNA, cell-free DNA; cfFrag, cfDNA fragmentomics; NAC, neoadjuvant chemotherapy; pCR, pathological complete response.

We enrolled a total of 613 female participants in this multi-center study. In the training set, we enrolled 91 patients with breast cancer and 102 women with breast benign nodules from the Yantai Yuhuangding Hospital, The Affiliated Hospital of Qingdao University (YYH) in Yantai, China (the Yantai cohort). Seven patients who refused to biopsy were excluded. To comprehensively evaluate the cfFrag assay, we enrolled five external validation cohorts for different clinical utilities. First, we prospectively recruited 143 patients with breast cancer and 66 women with benign nodules from the Cancer Hospital of the Chinese Academy of Medical Sciences (CHCAMS) in Beijing, China (the Beijing cohort). Second, we incorporated another independent validation cohort, including 40 female patients with breast cancer and 13 women with benign breast nodules from The Second Affiliated Hospital of Zhejiang University School of Medicine in Hangzhou, China (the Hangzhou cohort). Third, as the external screening cohort, we recruited 119 asymptomatic healthy women from our previous cohort of non-cancer healthy volunteers in Nanjing, China [14]. Next, the NAC validation cohort included 9/33 (27.3%) patients with pathological complete response (pCR) and 24/33 (72.7%) patients with non-pCR from the CHCAMS. Finally, the robustness analysis cohort contained three stage II breast cancer patients and three patients with benign nodules (**Figure 1**).

The breast cancer patients enrolled in the training and prospective validation cohorts (the Yantai and Beijing cohorts) were all in the early stages (0–II), including 8.8% and 16.0% in ductal carcinoma *in situ* (DCIS)/stage 0, 36.3% and 39.9% in stage I, and 54.9% and 42.0% in stage II (Table S1). Among these patients, the majority type of breast cancer (80.4% and 85.7%) was invasive ductal carcinoma (IDC), and 16.1% and 18.7% of them were identified as triple-negative breast cancer (TNBC) in both cohorts.

### Whole-genome multi-feature analysis of cfDNA identifies optimal cfFrag profiles for breast cancer detection

In the Yantai cohort (training set), an average amount of 5.6 ng cfDNA (2.3–26.5 ng) was extracted from 500 µl plasma. In the Beijing cohort (validation set), 2 ml plasma was used to extract cfDNA for an average amount of 8.8 ng (3.4–13.5 ng). We applied low-depth WGS to the cfDNA samples. Libraries were sequenced in 7.4× mean depth (2.9× to 11.3×) in the training set and 8.8× mean depth (3.4× to 13.5×) in the validation set, resulting in a highly unique mapping rate and unique deduplicated mapping rate of more than 99.96%. The cfFrag profiles were generated using low-depth WGS data from plasma cfDNA. To find optimal features for model construction, six types of cfFrag profiles, including copy number variation (CNV), fragment size distribution (FSD), fragment size ratio (FSR), Griffin, motif breakpoint (MBP), and neomer, were generated using in-house scripts as previously reported [13,15,20–23]. Distinct spectra of cfFrag features were found in patients with breast cancer and benign nodules, especially in CNV, FSD, and FSR (Figure S1).

We used the tumor fraction (TF) reported by ichorCNA [15] to show the differences in CNV profiles between breast cancer patients and benign nodule patients. As shown in Figure S2, the TF was significantly higher for the breast cancer patients than the benign nodule patients in both the training cohort (*P* = 8.0 × 10^−5^) and the validation cohort (*P* = 0.029). This suggests that while the breast cancer and benign groups both vary substantially from healthy baselines, there are still distinguishable differences between the two groups.

**Figure 2:**
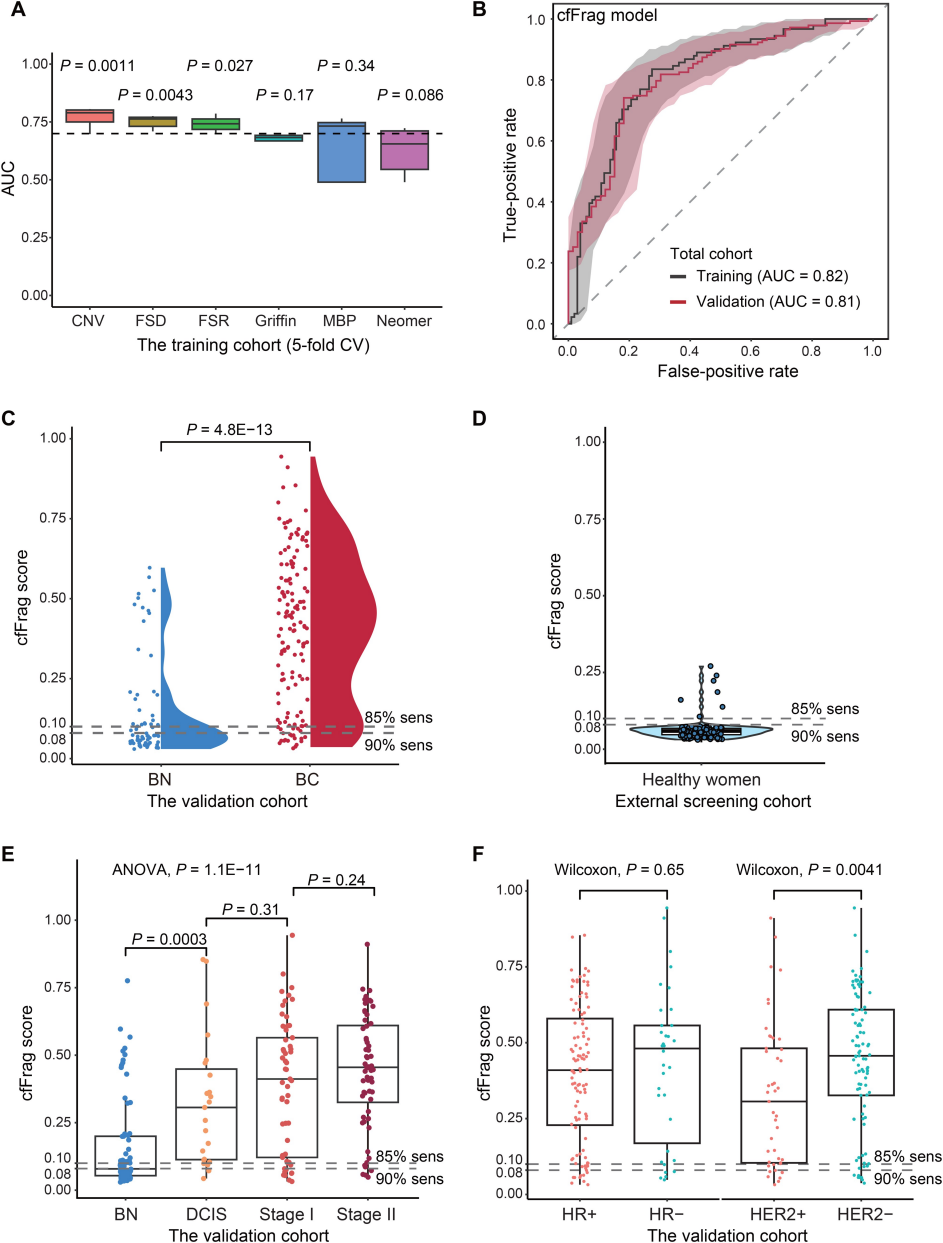
Evaluation of the cfFrag model **A**. Box plot for AUCs of top base learners for six different cfDNA fragmentomics features. *P* values of the six fragmentomics features *vs.* all features were determined by Student’s *t*-test. **B**. ROC curves using the training cohort (5-fold CV) and the independent validation cohort. **C**. Violin plot illustrating the cfFrag score distribution in the independent validation cohort’s benign nodule and breast cancer groups. The cut-off values, shown as the dotted lines, were determined by the training cohort. **D**. Violin plot illustrating the cfFrag score distribution in 119 healthy female volunteers from our previous study [13]. The cfFrag scores for most healthy women (112/119) were lower than both cut-off values, yielding an excellent specificity of 94.1%. **E**. Box plot illustrating the cfFrag score distribution in the benign nodule group as well as the very-early-stage (DCIS/stage 0) and early-stage (stages I and II) breast cancer groups. **F**. Box plot illustrating the cfFrag score distribution in different subgroups of the validation cohort. AUC, area under the curve; FSR, fragment size ratio; FSD, fragment size distribution; CNV, copy number variation; MBP, motif breakpoint; CV, cross-validation; sens, sensitivity; BN, benign nodule; BC, breast cancer; ROC, receiver operating characteristic; DCIS, ductal carcinoma *in situ*; HR, hormone receptor; HER2, human epidermal growth factor receptor 2; ANOVA, analysis of variance.

Next, base learners were constructed and optimized utilizing five different algorithms of the machine learning process [13] on the training set. Among the six fragmentomics features, CNV, FSD, and FSR demonstrated significantly higher area under the curve (AUC) values compared to all features (Student’s *t*-test, *P* = 1.1 × 10^−3^, *P* = 4.3 × 10^−3^, and *P* = 2.7 × 10^−2^, respectively) (**Figure 2**A).

### The cfFrag model accurately distinguishes early-stage breast cancer from benign nodules with high specificity in asymptomatic healthy women

The cfFrag scores were constructed using the optimal three cfFrag profiles (CNV, FSR, and FSD) to predict breast cancer in the training cohort. A total of 24 (3 × 8) top base learners were selected to create the final cfFrag score by the 5-fold cross-validation (CV) AUC in the training cohort (Figures S3 and S4). Among the three feature types, CNV showed the highest mean AUC of 0.742 (0.661–0.791) for its top 8 base learners, while the FSD and FSR showed similar predictive power in mean AUC [0.706 (0.631–0.750) and 0.706 (0.647–0.754), respectively] (Table S2). The top-performing features for each feature type were identified by summarizing the ranking of their relative importance in each base learner, as shown in Tables S3–S5.

To illustrate the impact of these top-performing features on the final cfFrag model, a recursive feature elimination analysis was performed. We constructed multiple cfFrag models using various subsets of top-performing features and evaluated their performance in the training and validation cohorts. The cfFrag model showed possible overfitting in the training cohort by using only subsets of top-performing features in the final model (Figure S5). The 5-fold CV AUCs in the training cohort gradually decreased as more features were used in the model construction process. The cfFrag model illustrated a solid discriminatory power between the breast cancer and benign nodules, yielding AUCs of 0.82 [95% confidence interval (CI): 0.76–0.88] and 0.81 (95% CI: 0.75–0.87) in training and external validation cohorts, respectively (Figure 2B).

The distribution patterns of the cfFrag scores showed significant differences between the benign nodule and breast cancer groups in the training cohort (Wilcoxon rank-sum test, *P* = 3.9 × 10^−14^) (Figure S6), suggesting that the cfFrag scores were positively associated with the probability of breast cancer. A similar trend was observed in the prospective validation cohort, with the breast cancer group showing a significantly higher cfFrag score than the benign nodule group (*P* = 4.8 × 10^−13^) (Figure 2C). It achieved a specificity of 51.5% (95% CI: 38.9%–64.0%) at the designed 89.5% sensitivity (95% CI: 83.3%–94%) in the independent validation cohort, resulting in an overall accuracy of 77.5% (95% CI: 71.2%–83.0%) (**Table 1**). Setting the cut-off value at 85% sensitivity, the cfFrag model reached specificities of 65.7% (95% CI: 55.6%–74.8%) and 60.6% (95% CI: 47.8%–72.4%) in the training and validation cohorts, respectively (Table S6).

**Table 1:** Performance evaluation of the cfFrag model in the training and validation cohorts

	Training cohort (5-fold cross-validation)	Prospective validation cohort	Asymptomatic healthy women
No. of patients with breast cancer	91	143	–
No. of patients with benign nodules	102	66	–
No. of healthy controls	–	–	119
Sensitivity (95% CI)	90.1% (82.1%–95.4%)	89.5% (83.3%–94%)	–
Specificity (95% CI)	52.0% (41.8%–62.0%)	51.5% (38.9%–64%)	94.1% (88.3%–97.6%)
PPV (95% CI)	62.6% (53.7%–70.9%)	80.0% (73.0%–85.9%)	–
NPV (95% CI)	85.5% (74.2%–93.1%)	69.4% (54.6%–81.7%)	–
Accuracy (95% CI)	69.9% (62.9%–76.3%)	77.5% (71.2%–83%)	–

*Note*: cfFrag, cell-free DNA fragmentomics; CI, confidence interval; PPV, positive predictive value; NPV, negative predictive value.

To further validate the performance of the cfFrag model and included features (CNV, FSR, and FSD) and to rule out the potential overfitting, we incorporated another independent validation cohort in Hangzhou, China. In this Hangzhou cohort, we newly enrolled 40 patients with breast cancer and 13 patients with benign breast nodules. Using a simplified model based on single features, the diagnostic AUCs achieved 0.806, 0.946, and 0.894 for CNV, FSR, and FSD, respectively, with a combined model AUC of 0.954 (Figure S7A). The cfFrag scores were significantly higher in patients with breast cancer than those with benign nodules, performing a specificity of 76.92% with a sensitivity of more than 90% (Figure S7B). The independent validation confirmed the robustness of the model and its included features and highlighted the dominant contribution of FSR.

To verify the specificity of the cfFrag score in healthy women, we analyzed the cfDNA WGS data from 119 asymptomatic healthy women to generate the cfFrag scores. As a result, it yielded an excellent specificity of 94.1% (112/119, 95% CI: 88.3%–97.6%) (Figure 2D; Table 1) for both cut-off values for 85% and 90% sensitivities.

### The cfFrag model maintains excellent performance in subgroup analysis and correlation with clinical features

To address the potential bias brought by the imbalanced age between breast cancer and benign nodule patients, a propensity score matching analysis was performed. We selected 112 patients (64 breast cancer patients and 48 benign nodule patients with matched ages) from the training cohort and 174 patients (117 breast cancer patients and 57 benign nodule patients with matched ages) from the prospective validation cohort. As a result, the cfFrag model showed equally excellent predictive ability for breast cancer in these age-matched subsets, yielding AUCs of 0.82 (95% CI: 0.73–0.90) and 0.82 (95% CI: 0.75–0.88) in the training and validation cohorts, respectively (Figure S8A). Similarly, the predictive model was able to maintain its predictive ability in a cohort containing small nodules (size ≤ 1 cm), showing a high AUC of 0.83 (95% CI: 0.72–0.95) in the validation cohort (Figure S8B), compared to the traditional imaging methods (AUC = 0.64 and AUC = 0.80 for mammography and ultrasound, respectively) (Figure S9).

A subgroup analysis focused on the model’s performance was also performed to investigate potential bias. The sensitivities remained high for detecting various breast cancer subgroups, including the nodule size, stage, histology, and hormone receptor (HR) status (Figure S10). As the size of the benign nodules increased, the ability to identify different subgroups with specificity decreased (< 2 cm: 54.9%, 2–5 cm: 40.0%) (Figure S11).

In addition to conducting subgroup analysis within the validation cohort, we performed a bootstrap analysis to minimize potential bias. Sensitivities derived from 100 bootstrap iterations for various breast cancer subgroups displayed patterns similar to our previous observations (Figure S12). Additionally, the specificities for the benign nodule subgroups, assessed through 100 bootstrap iterations, aligned with the trends seen in the validation cohort (Figure S13).

To demonstrate the performance for early detection, the cfFrag scores showed a significant gradual increase from the benign nodule to the DCIS and early-stage (stages I and II) breast cancer [analysis of variance (ANOVA), *P* = 1.1 × 10^−11^] (Figure 2E). Although the cfFrag score distribution showed no significant difference between the HR-positive and HR-negative groups, as well as between the TNBC and non-TNBC groups, the human epidermal growth factor receptor 2 (HER2)-negative group’s cfFrag scores were significantly higher than the HER2-positive group (Wilcoxon rank-sum test, *P* = 4.1 × 10^−3^) (Figure 2F, Figure S14). This suggested the potential relation between the cfFrag features and the molecular subtypes.

### The cfFrag model demonstrates robust performance in the downsampling process and high reproducibility between technical replicates

**Figure 3:**
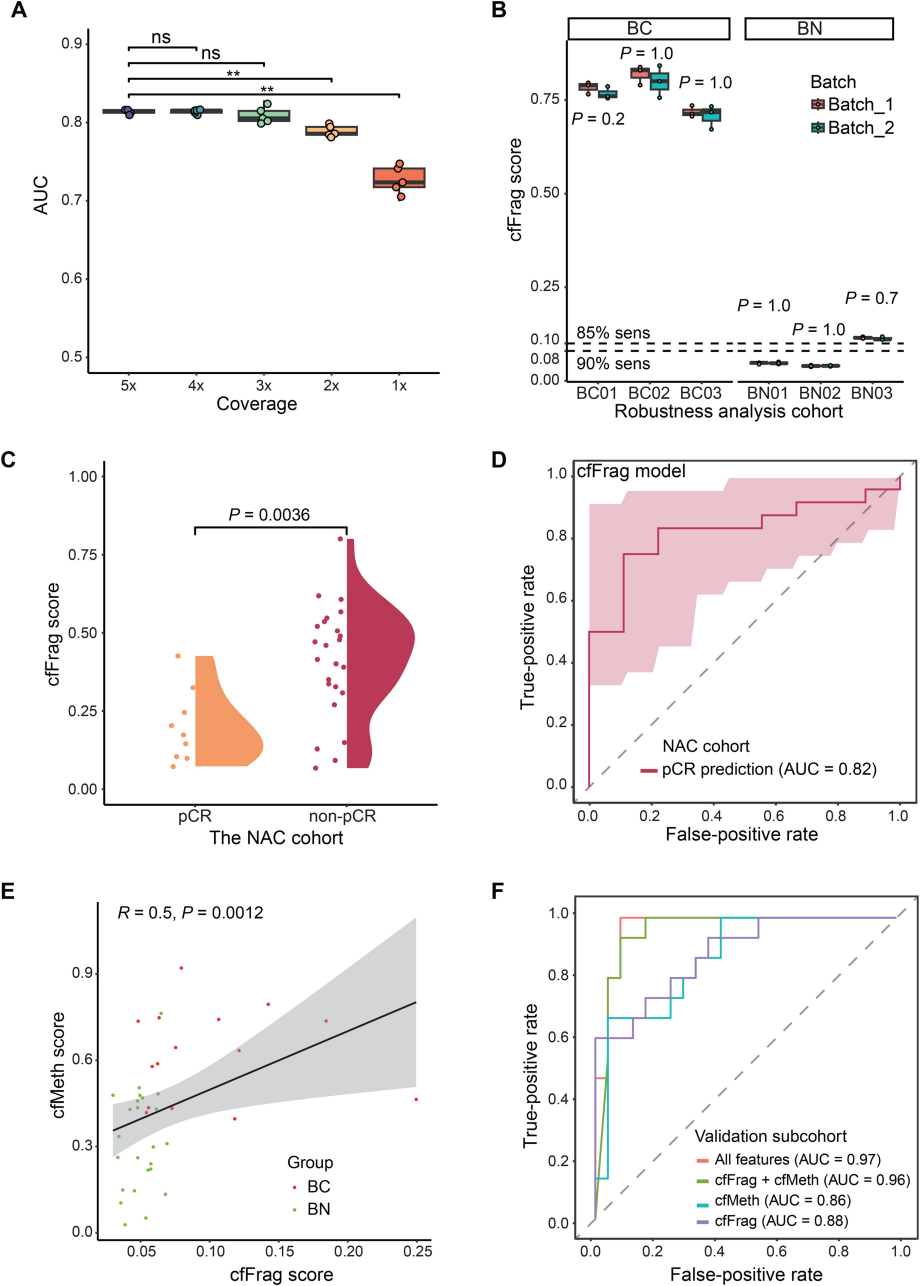
Comprehensive evaluation of the cfFrag model using additional cohorts **A**. Box plot for the validation cohorts’ AUCs for the downsampling process (5× to 1×). There is no significant performance drop till 3× coverage (ns, not significant; **, *P* < 0.01; Student’s *t*-test). **B**. Box plot for cfFrag scores in the external test cohort containing 3 breast cancer patients and 3 benign nodule patients. For each patient, 2 batches × 3 repeats were performed. **C**. Violin plot illustrating the cfFrag score distribution in patients with pCR and non-pCR. **D**. ROC curve for distinguishing patients with pCR (*N* = 9) from patients with non-pCR (*N* = 24) in a NAC cohort. **E**. Scatter plot showing the correlation between cfMeth scores and cfFrag scores for a subset of patients in the validation cohort (*N* = 39) with previously reported whole-genome bisulfite sequencing data. **F**. ROC curves for a subset of patients in the validation cohort (*N* = 39) with previously reported whole-genome bisulfite sequencing data and imaging data using leave-one-out CV. cfMeth, cfDNA methylation.

To decrease the potential cost and required blood samples in the future, we assessed the cfFrag model’s performance using downsampled WGS data (5× to 1×) in the validation cohort with five technical replicates generated for each coverage depth. The cfFrag model maintained its predictive power during the downsampling process without showing a significant decrease in AUCs, even at a depth of 3× (*P* > 0.05) (**Figure 3**A).

To assess the robustness of the cfFrag model within and between runs, two batches of 10 ml peripheral blood samples were collected from the six patients with a median interval of 5 days. The plasma samples were separated into three equal proportions as technical replicates for each batch, resulting in 36 samples. The model extracted and evaluated the cfFrag profiles of these 36 samples. All three cfFrag profiles (CNV, FSR, and FSD) showed no significant differences between the technical replicates and the two batches (Figure S15). Additionally, the robustness analysis showed no significant differences between runs and within runs (Wilcoxon rank-sum test, *P* = 0.2–1.0) (Figure 3B).

### The cfFrag model can predict the therapeutic pathological response for breast cancer patients after NAC

To expand the clinical utility of the cfFrag model to predict the treatment response, we applied this assay to 33 female breast cancer patients receiving the NAC. The cfFrag profiles were generated using post-NAC plasma samples and subsequently predicted by the cfFrag model. As a result, the cfFrag scores for pCR patients were significantly lower than the non-pCR patients (Wilcoxon rank-sum test, *P* = 3.6 × 10^−3^) (Figure 3C). However, it is noted that the cfFrag scores for the pCR patients were still higher than those for patients with benign nodules. Moreover, the cfFrag model demonstrated excellent performance in distinguishing between patients with pCR and with non-pCR, yielding an AUC of 0.82 (95% CI: 0.68–0.97) (Figure 3D). This indicates that the cfFrag model has the potential to predict the therapeutic response and minimal residual disease for post-NAC breast cancer patients.

### The fragmentomics and methylomics features of cfDNA exhibit complementarity in breast cancer detection

To investigate the potential use of combined WGS with whole-genome bisulfite sequencing (WGBS) data for the differentiating power of breast cancer and benign nodules. We selected 39 patients from the prospective validation cohort (including 15 breast cancer patients and 24 benign nodule patients) enrolled in a methylation-based breast cancer early detection analysis to generate the breast cancer risk score (the cfMeth score) through the WGBS [4]. We found that the cfFrag and cfMeth scores were positively correlated (Spearman’s rank correlation coefficient, *R* = 0.5, *P* = 1.2 × 10^−3^) (Figure 3E). Due to the limited size, leave-one-out cross-validation was performed. The combined (cfFrag + cfMeth) model showed better performance (AUC = 0.96, 95% CI: 0.89–1.00) than the cfFrag model alone (AUC = 0.88, 95% CI: 0.77–0.99) and the cfMeth model alone (AUC = 0.86, 95% CI: 0.75–0.98), while the addition of imaging data (cfFrag + cfMeth + X-ray + ultrasound) further improved the performance (AUC = 0.97, 95% CI: 0.92–1.00) (Figure 3F).

### The joint diagnostic model combining the cfFrag scores and breast imaging shows superior performance in detecting breast cancer

**Figure 4:**
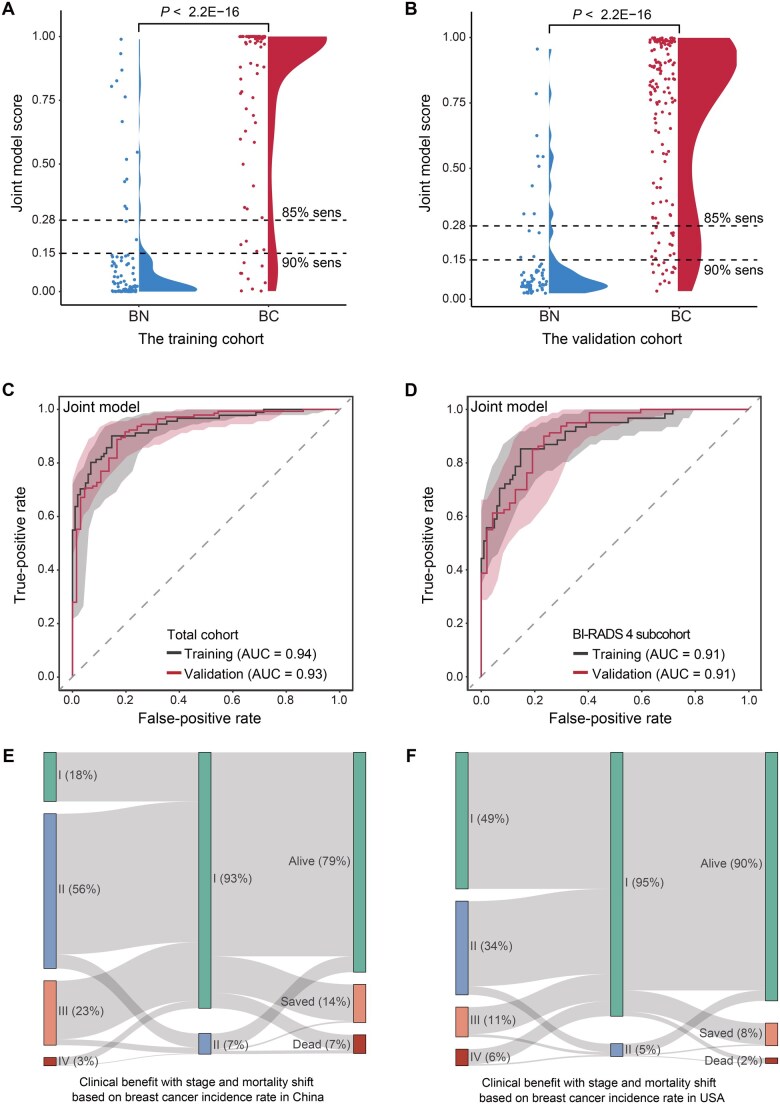
Performance evaluation of the joint model using both fragmentomics and imaging techniques **A**. Violin plot illustrating cancer score distribution of the joint model in the benign nodule and breast cancer groups in the training cohort. **B**. The score distribution of the joint model in the benign nodule and breast cancer groups in the independent validation cohort. **C**. ROC curves using the training cohort (5-fold CV) and the independent validation cohort. **D**. ROC curves for subset patients with BI-RADS category 4 in the training cohort (5-fold CV) and the validation cohort. **E**. Potential clinical benefit of the joint model using breast cancer statistics in China. **F**. Potential clinical benefit of the joint model using breast cancer statistics in the USA. The left bars show the current stage distributions of newly diagnosed breast cancer, and the middle bars indicate the stage distributions for potential clinical utilization of the joint model in the two countries. Accordingly, mortality shifts and 5-year survival benefits (orange bars) achieved by using the joint model are shown in the right bars. BI-RADS, breast imaging reporting and data system.

To further improve the performance of the cfFrag-based approach cost-effectively, a joint diagnostic model was constructed by integrating the cfFrag scores and the breast imaging reporting and data system (BI-RADS) categories for mammography and ultrasound using the machine learning process. As a result, the joint model risk score exhibited a significant difference between the breast cancer and benign nodule groups in both the training and validation cohorts (Wilcoxon rank-sum text, *P* < 2.2 × 10^−16^) (**Figure 4**A and B).

The joint diagnostic model showed superior performance for distinguishing breast cancer from benign nodules, with AUCs of 0.94 (95% CI: 0.90–0.97) and 0.93 (95% CI: 0.89–0.97) in the training and validation cohorts, respectively (Figure 4C), which was significantly higher than the cfFrag model alone, as well as the traditional mammography and ultrasound (all *P* < 0.05) (Figure S16). Furthermore, the joint model could reach a high specificity of 80.3% (95% CI: 68.7%–89.1%) at the designed 90.2% sensitivity (95% CI: 84.1%–94.5%) in the independent validation cohort (**Table 2**). Furthermore, the joint model maintained its performance within women with the BI-RADS 4 lesions, reaching AUCs of 0.91 (95% CI: 0.86–0.95) and 0.91 (95% CI: 0.86–0.96) in the training and validation cohorts, respectively (Figure 4D).

**Table 2:** Performance evaluation of the joint model in the training and validation cohorts

	Training cohort (5-fold cross-validation)	Prospective validation cohort
No. of patients with breast cancer	91	143
No. of patients with benign nodules	102	66
Sensitivity (95% CI)	90.1% (82.1%–95.4%)	90.2% (84.1%–94.5%)
Specificity (95% CI)	85.3% (76.9%–91.5%)	80.3% (68.7%–89.1%)
PPV (95% CI)	84.5% (75.8%–91.1%)	90.8% (84.9%–95.0%)
NPV (95% CI)	90.6% (82.9%–95.6%)	79.1% (67.4%–88.1%)
Accuracy (95% CI)	87.6% (82.1%–91.9%)	87.1% (81.8%–91.3%)

### The joint model demonstrates potential for enhancing early detection rates and improving survival outcomes in China and the USA

To assess the potential clinical benefits of the joint model in a real-world setting, we utilized an intercept model developed by Hubbell and his colleagues [24]. Currently, only 18% of breast cancer patients are diagnosed at stage I in China. By utilizing the joint model, the detection rate of stage I breast cancer could be elevated to 93%. Accordingly, less breast cancer would be diagnosed at stages II–IV. Based on the stage shifts, it was estimated that the joint model could increase the 5-year survival rates of breast cancer in China by 14% (Figure 4E). Similarly, in the USA, the increased detection rate of stage I breast cancer (95%) and the 5-year survival benefit (8%) were also estimated (Figure 4F).

## Discussion

Current imaging-based breast cancer screening methods suffer from high false positives and inconclusive results among female patients with benign breast nodules, which leads to intrusive biopsy and unnecessarily adds to discomfort. In our study, we provided a highly sensitive, non-invasive diagnostic tool for early-stage breast cancer detection through the blood-based cfFrag analysis, especially against control patients with radiographically malignant-suspicious yet pathologically benign breast nodules. Notably, we have demonstrated significant complementarity between cfFrag, traditional imaging, and cfDNA methylation features in the early detection of breast cancer and the assessment of the benign or malignant nature of breast nodules. The combination of cfFrag and traditional imaging findings, as well as the combination of cfFrag and cfDNA methylation features, can further enhance the diagnostic accuracy of breast cancer, aligning with our previous findings on combining cfDNA methylation and traditional imaging in breast cancer. The joint diagnostic model, integrating our non-invasive cfFrag assay with image findings, achieves high diagnostic accuracy (AUC = 0.93–0.94). Accordingly, the joint model can guide biopsy decisions and reduce unnecessary invasive interventions by 80.3%–85.3% in patients with suspicious imaging results.

The detection rate/sensitivity is crucial to avoid cancer diagnostic delay. Thus, 85%–90% sensitivities for early-stage breast cancer were set as the primary endpoint for the cfFrag and joint models in this study. The cfFrag-only and joint models performed robust detection rates in early-stage breast cancer, even in women with small nodules or inconclusive imaging results (BI-RADS 4 lesions). Due to the potential stage shift and increase in the stage I breast cancer diagnosis rate, our joint model was estimated to save a significant number of breast cancer patients (an extra 8% and 14%) in the USA and China. However, further real-world studies are still needed to identify the cut-off value for each model with the best cost-effectiveness in different populations.

The detection rates were robustly elevated across increasing breast cancer stages in this study. Intriguingly, the cfFrag signal was more significant in patients with DCIS than those with stage I breast cancer, which is consistent with our methylation-based cfDNA analysis [4] but inconsistent with previous mutation-based cfDNA analysis [25]. It indicates the advantages of epigenetics-based and fragmentomics-based cfDNA analyses in the early detection of DCIS/stage 0 breast cancer. In addition, the cfFrag model has shown sufficient specificity in asymptomatic healthy women, further indicating the potential clinical utility of the cfFrag model in population-based breast cancer screening.

The use of peripheral cfDNA has gained prominence in early cancer detection, as methylation-based and fragmentomics-based cfDNA markers have demonstrated effectiveness in detecting many cancer types [4,13–15,26]. Methylation features in cfDNA are related to the cancer tissue-of-origin, while cfFrag features are linked to the abnormal DNA nuclease activities in cancers [27]. Compared to the methylation-based cfDNA approach, fragmentomics-based cfDNA assays offer advantages such as lower cost by avoiding sodium bisulfite treatment and requiring less blood sample volume because of low sequence depth. Interestingly, our combined analysis of cfDNA methylation and fragmentomics features reveals that combining the fragmentomics (cfFrag score) and the methylation markers (cfMeth score) could achieve superior performance than each marker separately, which was in agreement with the result of the recent sub-study in the Circulating Cell-free Genome Atlas [28].

Monitoring treatment response is crucial to deciding the subsequent treatment strategies for breast cancer patients receiving NAC, but this was unmet using the current methods [29]. Recently, mutation-based and methylation-based ctDNA detection approaches have been demonstrated to predict the treatment response and residual disease in post-NAC breast cancer patients [30,31]. Our study suggests that the features of cfFrag could be used as an alternative approach to evaluate the treatment response in breast cancer patients.

Our study has two main limitations. Firstly, the sample size of the combined analysis of the cfFrag and methylation is relatively small. Multi-omics cfDNA analysis with large sample sizes is still needed to identify the optimal non-invasive combination with low cost using a trace amount of blood sample for the early detection of breast cancer. Secondly, similar to most previous cfFrag studies that only focused on one cancer type [13–15], we also aimed to identify the breast cancer-specific cfFrag features in this study. With the identification of the cfFrag spectra in different cancer types in the future, it would be cost-effective to develop a pan-cancer diagnostic model to detect multiple types of cancer and indicate the cancer origin.

## Conclusion

This pilot study systematically evaluates the performance of cfFrag as a non-invasive biomarker for breast cancer. The low-depth cfFrag profiling with automated machine learning demonstrated excellent and robust performance in distinguishing early-stage breast cancer from benign nodules with inconclusive imaging results, predicting NAC response, and maintaining sufficient specificity in asymptomatic healthy women in a multi-center prospective setting. The combination of non-invasive cfFrag features and standard diagnostic imaging improved the detection accuracy of early breast cancer. This approach holds promise for improving clinical outcomes and streamlining healthcare practices.

## Materials and methods

### Study design and participants

In this multi-center study, we recruited female patients independently in four centers in China. The training set enrolled 200 consecutive female patients with malignant-suspicious breast imaging results from the YYH in Yantai (the Yantai cohort). The external validation sets prospectively enrolled 209 consecutive female patients who underwent breast lesion biopsy (the Beijing cohort), 33 female breast cancer patients after NAC (the NAC validation cohort), and six female patients with repeating samples for robustness analysis (the robustness validation cohort) from the CHCAMS in Beijing. The external screening cohort recruited 119 asymptomatic healthy women from our previous Nanjing cohort [14]. We also incorporated an independent validation cohort from Hangzhou (the Hangzhou cohort) to evaluate each included complementary DNA (cDNA) feature. The recruitment period was from January 1, 2019 to August 1, 2022. This study adhered to the guidelines of the Standards for Reporting of Diagnostic Accuracy Studies (STARD).

### Sample collection and clinical evaluation

We collected 10 ml of peripheral blood samples from each participant before biopsy or surgery. In the Yantai cohort (training set), the collected blood samples were kept in ethylene diamine tetraacetic acid (EDTA) blood collection tubes (Catalog No. 367525, Becton Dickinson, Franklin Lakes, NJ) at 4°C and underwent centrifugations (1800 *g* for 10 min and 16,000 *g* for 10 min both at 4°C) within 2 h. In the Beijing cohort (independent validation set), the collected blood samples were kept in the Cell-Free DNA BCT blood collection tubes (Catalog No. 218997, Streck, Omaha, NE) at room temperature (RT, 15°C–25°C). Plasma was extracted within 48 h following blood collection by centrifugations of the blood according to the protocols in the training set.

Standard mammography and ultrasonography techniques were conducted at two centers and independently interpreted according to the BI-RADS standard. Patients with suspicious breast imaging results underwent surgical or core needle biopsies. The pathological examination of tissue specimens confirmed the malignant or benign status of each participant. Women with negative imaging or biopsy results were excluded from having breast cancer after a 6-month follow-up. The molecular subtype of each lesion was determined according to the pathologic criteria for HR (including estrogen receptor and progesterone receptor) and HER2 [32].

### Library preparation and WGS

For cfDNA extraction, we used the liquid handling platform (Microlab STAR, Hamilton Company, Reno, NV) and the QIAamp Circulating Nucleic Acid Kit (Catalog No. 55114, QIAGEN, Hamburg, Germany) according to previously reported protocols [13]. The Qubit dsDNA HS Assay Kit (Catalog No. Q32854, Thermo Fisher Scientific, Waltham, MA) was then utilized for measuring the concentration of the extracted cfDNA. The polymerase chain reaction (PCR)-free WGS library was automatically constructed on Biomek (Catalog No. Bimek i5, Beckman Coulter, Manchester, UK), using 5–10 ng of cfDNA sample and the KAPA Hyper Prep Kit (Catalog No. KK8504, KAPA Biosystems, Wilmington, MA). The constructed library was quantified by the KAPA SYBR FAST qPCR Master Mix (Catalog No. KK4824, KAPA Biosystems) before paired-end sequencing on NovaSeq platform (NovaSeq 6000Dx-CN, Illumina, San Diego, CA).

For the quality control of bioinformatics analysis, Trimmomatic [33] was used to trim the raw sequencing data. The removal of PCR duplicates was performed by the Picard toolkit (http://broadinstitute.github.io/picard/). The high-quality reads were then mapped to the human reference genome [Genome Reference Consortium Human37 (GRCh37) or University of California, Santa Cruz human genome19 (UCSC hg19)] using BWA sequence aligner [34].

### Fragmentomics profiling

As tumor cell fragments are shorter than those from normal cells [35], the FSR profile analyzed the ratio of short fragments in the human genome. Short fragments were defined as 100–150 bp and long fragments were defined as 151–220 bp [13,15,20]. The human genome was divided into 5-Mb bins, in which the ratios of short to long fragments were calculated, resulting in a total of 1082 (541 bins × 2) FSR features. The FSD profile focused on the detailed length patterns of cfDNA fragments, categorizing these fragments based on increments of 5 bp in the range of 100 bp to 220 bp [13,36]. The proportion of fragments in each bin was computed on the human chromosome arm level for human autosomes, resulting in 936 FSD features that machine learning algorithms can employ. The CNV profile was calculated using ichorCNA [15]. For each sample, the genome was divided into 1-Mb bins. The depth for each bin was then compared to the default baseline using the hidden Markov model (HMM). The log_2_ ratio for each bin was calculated, generating 2475 features. The profiling of Griffin, neomer, and MBP is present in File S1.

### cfFrag model construction and validation

A machine learning process that utilizes five different algorithms, including random forest (RF), generalized linear model (GLM), deep learning (DL), gradient boosting machine (GBM), and extreme gradient boosting (XGBoost) [13], was employed to generate optimal base learners. A breast cancer prediction model, namely the cfFrag scores, was developed using the mean value of top base learners ranked by the AUC of the 5-fold CV for the optimal three cfFrag profiles in the training cohort (the Yantai cohort; File S1).

A similar automated machine learning process was used to construct the joint diagnostic model by using the cfFrag scores as numeric features and the BI-RADS by mammography and ultrasound as categorical features. The process utilized a randomized search for automatic algorithm selection and hyperparameter tuning. The best-performing model was selected from a total of 200 trained models based on the highest AUC using the training cohort via a 5-fold CV approach. Cut-off values were determined using a 5-fold CV to predict the score of the training cohort to reach 85% and 90% sensitivities. The external independent validation cohorts evaluated the joint diagnostic model’s performance. In addition, to expand the clinical utility of the cfFrag model, its performance was further evaluated in the NAC cohort.

### Statistical analysis

The receiver operating characteristic (ROC) curves were generated by the pROC package (v1.17.0.1) [37]. The sensitivity, specificity, and accuracy with the corresponding 95% CI were calculated by the epiR package (v2.0.19; https://cran.r-universe.dev/epiR). Propensity score matching analysis used the MatchIt package (v4.2.0) [38]. All statistical analyses, including Student’s *t*-test, Wilcoxon rank-sum test, and ANOVA, were performed in R (v3.6.3; https://www.r-project.org/).

## Ethical statement

This study was reviewed and approved by the ethics committees of each center (Approval Nos. 22/291-3493 for the CHCAMS and 2020-289 for the YYH), China. Each participant provided written informed consent.

## Code availability

The analytic code is provided in open-source repositories in GitHub (https://github.com/cancer-oncogenomics/Detecting-Breast-Cancer-Through-cfDNA-Fragmentomics). The code has also been submitted to BioCode at the National Genomics Data Center (NGDC), China National Center for Bioinformation (CNCB) (BioCode: BT007588), which is publicly accessible at https://ngdc.cncb.ac.cn/biocode/tool/7588.

## Supplementary Material

qzaf028_Supplementary_Data

## Data Availability

Raw data from the deidentified participants generated in this study have been deposited in the Genome Sequence Archive for Human [39] at the NGDC, CNCB (GSA-Human: HRA008957), and are publicly accessible at https://ngdc.cncb.ac.cn/gsa-human.

## CRediT author statement

**Jiaqi Liu:** Conceptualization, Methodology, Investigation, Data curation, Formal analysis, Funding acquisition, Writing – original draft. **Yalun Li:** Investigation, Data curation, Validation, Writing – original draft. **Wanxiangfu Tang:** Methodology, Investigation, Data curation, Formal analysis, Writing – original draft. **Tianyi Qian:** Investigation, Data curation, Validation, Writing – review & editing. **Lijun Dai:** Methodology, Investigation, Writing – original draft. **Ziqi Jia:** Investigation, Data curation, Formal analysis, Writing – original draft. **Heng Cao:** Investigation, Data curation. **Chenghao Li:** Methodology, Investigation, Data curation. **Yuchen Liu:** Investigation, Data curation. **Yansong Huang:** Investigation, Data curation. **Jiang Wu:** Investigation, Data curation. **Dongxu Ma:** Investigation, Data curation. **Guangdong Qiao:** Investigation, Validation, Data curation. **Hua Bao:** Methodology, Investigation, Data curation. **Shuang Chang:** Methodology, Investigation, Data curation. **Dongqin Zhu:** Methodology, Investigation, Data curation. **Shanshan Yang:** Methodology, Investigation, Data curation. **Xuxiaochen Wu:** Methodology, Investigation, Data curation. **Xue Wu:** Methodology, Investigation, Data curation. **Hengyi Xu:** Investigation, Data curation. **Hongyan Chen:** Writing – review & editing, Supervision. **Yang Shao:** Methodology, Investigation, Supervision. **Xiang Wang:** Conceptualization, Supervision, Writing – review & editing. **Zhihua Liu:** Conceptualization, Supervision, Project administration, Writing – review & editing. **Jianzhong Su:** Conceptualization, Methodology, Investigation, Data curation, Formal analysis, Resources, Supervision, Project administration, Writing – original draft, Writing – review & editing. All authors have read and approved the final manuscript.

## ORCID

0000-0002-9775-2342 (Jiaqi Liu)

0000-0002-9055-1935 (Yalun Li)

0000-0002-9045-2478 (Wanxiangfu Tang)

0000-0001-6532-7504 (Tianyi Qian)

0009-0004-2079-9427 (Lijun Dai)

0000-0002-1480-9498 (Ziqi Jia)

0000-0001-5985-3364 (Heng Cao)

0000-0002-2768-265X (Chenghao Li)

0000-0003-2698-3482 (Yuchen Liu)

0009-0000-3149-3440 (Yansong Huang)

0000-0002-6374-3077 (Jiang Wu)

0000-0001-5142-6667 (Dongxu Ma)

0000-0002-5298-3411 (Guangdong Qiao)

0000-0001-5774-8755 (Hua Bao)

0009-0003-0749-0372 (Shuang Chang)

0000-0002-8276-9109 (Dongqin Zhu)

0009-0005-0951-5179 (Shanshan Yang)

0009-0006-9529-871X (Xuxiaochen Wu)

0000-0002-5567-1325 (Xue Wu)

0000-0002-5772-0661 (Hengyi Xu)

0000-0003-4924-6100 (Hongyan Chen)

0000-0003-4585-1792 (Yang Shao)

0000-0002-1522-9532 (Xiang Wang)

0000-0003-4242-033X (Zhihua Liu)

0000-0003-1054-6042 (Jianzhong Su)

## References

[1] Pace LE , Keating NL. A systematic assessment of benefits and risks to guide breast cancer screening decisions. JAMA 2014;311:1327–35. 24691608 10.1001/jama.2014.1398

[2] Giaquinto AN , Sung H, Miller KD, Kramer JL, Newman LA, Minihan A, et al Breast cancer statistics, 2022. CA Cancer J Clin 2022;72:524–41. 36190501 10.3322/caac.21754

[3] Bevers TB , Helvie M, Bonaccio E, Calhoun KE, Daly MB, Farrar WB, et al Breast Cancer Screening and Diagnosis, version 3.2018, NCCN clinical practice guidelines in oncology. J Natl Compr Canc Netw 2018;16:1362–89. 30442736 10.6004/jnccn.2018.0083

[4] Liu J , Zhao H, Huang Y, Xu S, Zhou Y, Zhang W, et al Genome-wide cell-free DNA methylation analyses improve accuracy of non-invasive diagnostic imaging for early-stage breast cancer. Mol Cancer 2021;20:36. 33608029 10.1186/s12943-021-01330-w PMC7893735

[5] Corcoran RB , Chabner BA. Application of cell-free DNA analysis to cancer treatment. N Engl J Med 2018;379:1754–65. 30380390 10.1056/NEJMra1706174

[6] Bidard FC , Hardy-Bessard AC, Dalenc F, Bachelot T, Pierga JY, de la Motte Rouge T, et al Switch to fulvestrant and palbociclib *versus* no switch in advanced breast cancer with rising *ESR1* mutation during aromatase inhibitor and palbociclib therapy (PADA-1): a randomised, open-label, multicentre, phase 3 trial. Lancet Oncol 2022;23:1367–77. 36183733 10.1016/S1470-2045(22)00555-1

[7] Henry NL , Somerfield MR, Dayao Z, Elias A, Kalinsky K, McShane LM, et al Biomarkers for systemic therapy in metastatic breast cancer: ASCO guideline update. J Clin Oncol 2022;40:3205–21. 35759724 10.1200/JCO.22.01063

[8] Liu J , Huang Y, Wang X. Mutation-based circulating tumor DNA detection approach for monitoring the therapy response in breast cancer. J Natl Cancer Cent 2023;3:254–5. 39036666 10.1016/j.jncc.2023.08.006 PMC11256518

[9] Alix-Panabières C , Pantel K. Liquid biopsy: from discovery to clinical application. Cancer Discov 2021;11:858–73. 33811121 10.1158/2159-8290.CD-20-1311

[10] Cohen JD , Li L, Wang Y, Thoburn C, Afsari B, Danilova L, et al Detection and localization of surgically resectable cancers with a multi-analyte blood test. Science 2018;359:926–30. 29348365 10.1126/science.aar3247 PMC6080308

[11] Gianni C , Palleschi M, Merloni F, Di Menna G, Sirico M, Sarti S, et al Cell-free DNA fragmentomics: a promising biomarker for diagnosis, prognosis and prediction of response in breast cancer. Int J Mol Sci 2022;23:14197. 36430675 10.3390/ijms232214197 PMC9695769

[12] Wu SL , Zhang X, Chang M, Huang C, Qian J, Li Q, et al Genome-wide 5-hydroxymethylcytosine profiling analysis identifies MAP7D1 as a novel regulator of lymph node metastasis in breast cancer. Genomics Proteomics Bioinformatics 2021;19:64–79. 33716151 10.1016/j.gpb.2019.05.005 PMC8498923

[13] Bao H , Wang Z, Ma X, Guo W, Zhang X, Tang W, et al Letter to the editor: an ultra-sensitive assay using cell-free DNA fragmentomics for multi-cancer early detection. Mol Cancer 2022;21:129. 35690859 10.1186/s12943-022-01594-w PMC9188251

[14] Wang S , Meng F, Li M, Bao H, Chen X, Zhu M, et al Multidimensional cell-free DNA fragmentomic assay for detection of early-stage lung cancer. Am J Respir Crit Care Med 2023;207:1203–13. 36346614 10.1164/rccm.202109-2019OC PMC10161762

[15] Ma X , Chen Y, Tang W, Bao H, Mo S, Liu R, et al Multi-dimensional fragmentomic assay for ultrasensitive early detection of colorectal advanced adenoma and adenocarcinoma. J Hematol Oncol 2021;14:175. 34702327 10.1186/s13045-021-01189-w PMC8549237

[16] Moldovan N , van der Pol Y, van den Ende T, Boers D, Verkuijlen S, Creemers A, et al Multi-modal cell-free DNA genomic and fragmentomic patterns enhance cancer survival and recurrence analysis. Cell Rep Med 2024;5:101349. 38128532 10.1016/j.xcrm.2023.101349 PMC10829758

[17] Liu D , Yehia L, Dhawan A, Ni Y, Eng C. Cell-free DNA fragmentomics and second malignant neoplasm risk in patients with PTEN hamartoma tumor syndrome. Cell Rep Med 2024;5:101384. 38242121 10.1016/j.xcrm.2023.101384 PMC10897513

[18] Helzer KT , Sharifi M, Sperger JM, Shi Y, Annala M, Bootsma ML, et al Fragmentomic analysis of circulating tumor DNA targeted cancer panels. Ann Oncol 2023;34:813–25. 37330052 10.1016/j.annonc.2023.06.001 PMC10527168

[19] Wu N , Zhang Z, Zhou X, Zhao H, Ming Y, Wu X, et al Mutational landscape and genetic signatures of cell-free DNA in tumour-induced osteomalacia. J Cell Mol Med 2020;24:4931–43. 32277576 10.1111/jcmm.14991 PMC7205804

[20] Cristiano S , Leal A, Phallen J, Fiksel J, Adleff V, Bruhm DC, et al Genome-wide cell-free DNA fragmentation in patients with cancer. Nature 2019;570:385–9. 31142840 10.1038/s41586-019-1272-6 PMC6774252

[21] Georgakopoulos-Soares I , Barnea OY, Mouratidis I, Chan C, Bradley R, Mahajan M, et al Leveraging sequences missing from the human genome to diagnose cancer. medRxiv 2021;21261805. 10.1038/s43856-025-01067-3 PMC12371106 40841759

[22] Doebley AL , Ko M, Liao H, Cruikshank AE, Santos K, Kikawa C, et al A framework for clinical cancer subtyping from nucleosome profiling of cell-free DNA. Nat Commun 2022;13:7475. 36463275 10.1038/s41467-022-35076-w PMC9719521

[23] Guo W , Chen X, Liu R, Liang N, Ma Q, Bao H, et al Sensitive detection of stage I lung adenocarcinoma using plasma cell-free DNA breakpoint motif profiling. EBioMedicine 2022;81:104131. 35780566 10.1016/j.ebiom.2022.104131 PMC9251329

[24] Hubbell E , Clarke CA, Aravanis AM, Berg CD. Modeled reductions in late-stage cancer with a multi-cancer early detection test. Cancer Epidemiol Biomarkers Prev 2021;30:460–8. 33328254 10.1158/1055-9965.EPI-20-1134

[25] Chin YM , Takahashi Y, Chan HT, Otaki M, Fujishima M, Shibayama T, et al Ultradeep targeted sequencing of circulating tumor DNA in plasma of early and advanced breast cancer. Cancer Sci 2021;112:454–64. 33075187 10.1111/cas.14697 PMC7780051

[26] Xu RH , Wei W, Krawczyk M, Wang W, Luo H, Flagg K, et al Circulating tumour DNA methylation markers for diagnosis and prognosis of hepatocellular carcinoma. Nat Mater 2017;16:1155–61. 29035356 10.1038/nmat4997

[27] Zhou Z , Ma ML, Chan RWY, Lam WKJ, Peng W, Gai W, et al Fragmentation landscape of cell-free DNA revealed by deconvolutional analysis of end motifs. Proc Natl Acad Sci U S A 2023;120:e2220982120. 37075072 10.1073/pnas.2220982120 PMC10151549

[28] Jamshidi A , Liu MC, Klein EA, Venn O, Hubbell E, Beausang JF, et al Evaluation of cell-free DNA approaches for multi-cancer early detection. Cancer Cell 2022;40:1537–49.e12. 36400018 10.1016/j.ccell.2022.10.022

[29] Graeser M , Schrading S, Gluz O, Strobel K, Würstlein R, Kümmel S, et al Early response by MR imaging and ultrasound as predictor of pathologic complete response to 12-week neoadjuvant therapy for different early breast cancer subtypes: combined analysis from the WSG ADAPT subtrials. Int J Cancer 2021;148:2614–27. 33533487 10.1002/ijc.33495 PMC8048810

[30] Magbanua MJM , Brown Swigart L, Ahmed Z, Sayaman RW, Renner D, Kalashnikova E, et al Clinical significance and biology of circulating tumor DNA in high-risk early-stage HER2-negative breast cancer receiving neoadjuvant chemotherapy. Cancer Cell 2023;41:1091–102.e4. 37146605 10.1016/j.ccell.2023.04.008 PMC10330514

[31] Moss J , Zick A, Grinshpun A, Carmon E, Maoz M, Ochana BL, et al Circulating breast-derived DNA allows universal detection and monitoring of localized breast cancer. Ann Oncol 2020;31:395–403. 32067681 10.1016/j.annonc.2019.11.014

[32] Waks AG , Winer EP. Breast cancer treatment: a review. JAMA 2019;321:288–300. 30667505 10.1001/jama.2018.19323

[33] Bolger AM , Lohse M, Usadel B. Trimmomatic: a flexible trimmer for Illumina sequence data. Bioinformatics 2014;30:2114–20. 24695404 10.1093/bioinformatics/btu170 PMC4103590

[34] Li H , Durbin R. Fast and accurate short read alignment with Burrows–Wheeler transform. Bioinformatics 2009;25:1754–60. 19451168 10.1093/bioinformatics/btp324 PMC2705234

[35] Jiang P , Chan CW, Chan KC, Cheng SH, Wong J, Wong VW, et al Lengthening and shortening of plasma DNA in hepatocellular carcinoma patients. Proc Natl Acad Sci U S A 2015;112:E1317–25. 25646427 10.1073/pnas.1500076112 PMC4372002

[36] Zhang X , Wang Z, Tang W, Wang X, Liu R, Bao H, et al Ultrasensitive and affordable assay for early detection of primary liver cancer using plasma cell-free DNA fragmentomics. Hepatology 2022;76:317–29. 34954829 10.1002/hep.32308

[37] Robin X , Turck N, Hainard A, Tiberti N, Lisacek F, Sanchez JC, et al pROC: an open-source package for R and S+ to analyze and compare ROC curves. BMC Bioinformatics 2011;12:77. 21414208 10.1186/1471-2105-12-77 PMC3068975

[38] Ho D , Imai K, King G, Stuart EA. MatchIt: nonparametric preprocessing for parametric causal inference. J Stat Softw 2011;42:1–28.

[39] Chen T , Chen X, Zhang S, Zhu J, Tang B, Wang A, et al The Genome Sequence Archive Family: toward explosive data growth and diverse data types. Genomics Proteomics Bioinformatics 2021;19:578–83. 34400360 10.1016/j.gpb.2021.08.001 PMC9039563

