# Supplementary material for: Cell-free DNA Fragmentomics Assay to Discriminate the Malignancy of Breast Nodules and Evaluate Treatment Response
Source: Genomics Proteomics Bioinformatics. 2025 Apr 4;23(2):qzaf028. doi: 10.1093/gpbjnl/qzaf028 (PMC12321295; doi:10.1093/gpbjnl/qzaf028)
Supplement: qzaf028_Supplementary_Data [file qzaf028_supplementary_data.zip › supplementary material captions.docx]

# Supplemental material

**File S1 supplementary methods**

# Figure S1 Multi-omics cell-free DNA profiles of BC patients, and subjects with BN

**A.** Frequencies of chromosome arm-level CNVs in subjects with BC and BN. Amplifications are represented in red, while losses are depicted in blue. **B.** FSD in chromosome 18p across various groups. The distribution illustrates the fragment size profiles among subjects with BN and BC. **C.** Ratio of short (100–150 bp) fragments to long (150–220 bp) fragments across all 5 Mb bins on chromosomal arms in subjects with BC and BN.

# Figure S2 The ichorCNA TF distribution of BC and BN in the training cohort and the validation cohort

We used the ichorCNA reported TF to show the differences in CNV profile between BC patients and BN patients. The TF reported by ichorCNA was significantly higher for the BC patients compared to the BN patients in both the training cohort (*P* = 8 × 10^−5^) and the validation cohort (*P* = 0.029). This suggests that while the BC and BN groups both vary substantially from health baselines, there are still distinguishable differences between the two groups. TF, tumor fraction.

# Figure S3 The AUC distribution of base learners in the training cohort

A total of 24 (3 × 8) top base learners were selected to create the final cfFrag score by the 5-fold CV AUC in the training cohort.

# Figure S4 ROC curves for selected base learners in the training cohort

Among the three feature types, CNV showed the highest mean AUC of 0.742 (0.661–0.791) for its top 8 base learners, while the FSD and FSR showed similar predict power in mean AUC [0.706 (0.631–0.750) and 0.706 (0.647–0.754)].

# Figure S5 Feature recursive feature elimination analysis in the training cohort and the validation cohort

The cfFrag model showed possible overfitting in the training cohort using only subsets of top-performing features in the final model. The 5-fold CV AUCs in the training cohort showed a gradual decrease as more features were used in the model construction process.

# Figure S6 The cfFrag score distribution in the training cohort (5-fold CV)

# Figure S7 Performance evaluation for the base models (CNV, FSR, and FSD) and cfFrag model in the Hangzhou cohort

**A.** ROC curves of base models and cfFrag model in the Hangzhou cohort. The diagnostic AUCs were 0.806, 0.946, and 0.894 for CNV, FSR, and FSD, respectively, with a combined model AUC of 0.954. **B.** Distribution of cfFrag scores for BC and BN in the Hangzhou cohort.

# Figure S8 ROC curve of the cfFrag model in subsets of age-matched patients and patients with small nodule (≤ 1 cm) in the training and validation cohorts

**A.** ROC curves of the cfFrag model using an age-matched subset in the training cohort (5-fold CV) and the independent validation cohort. **B.** ROC curves of the cfFrag model using an subset of patients with small nodule (≤ 1 cm) in the training cohort (5-fold CV) and the independent validation cohort. The shadow areas indicate the 95% CI.

# Figure S9 ROC curves for traditional imaging technique in different cohorts

# Figure S10 Performance evaluation for fragmentomics model in different subgroups of BC patients in the validation cohort

IDC, invasive ductal carcinoma; TNBC, triple-negative breast cancer.

# Figure S11 Performance evaluation for fragmentomics model in different subgroups of patients with BN in the validation cohort

# Figure S12 Bootstrapped (100 times) performance evaluation of fragmentomics model in BC subgroups in the validation cohort

The sensitivities derived from 100 bootstrap iterations for various BC subgroups displayed patterns similar to our observations in Figure S5.

# Figure S13 Bootstrapped (100 times) performance evaluation of fragmentomics model in BN subgroups in the validation cohort

The specificities for the BN subgroup, assessed through 100 bootstrap iterations, align with the trends seen in the validation cohort (Figure S6).

# Figure S14 The cfFrag score distribution of different subgroups in the validation cohort (HR, HER2, and TNBC)

# Figure S15 The feature correlation of inter-runs and intra-runs

**A.**–**C.** No significant differences were observed between the technical replicates and the two batches in all three fragmentomics profiles, including CNV (A), FSD (B), and FSR (C).

# Figure S16 Comparing the joint model against the fragmentomics model, mammography, and ultrasound

**A.** and **B**. ROC curves for the training cohort (A; 5-fold CV) and the prospective validation cohort (B). The Wilcoxon *P* values compare the joint model against each individual technique.

**Table S1 Patient characteristics**

**Table S2 Selected top-performing base learners for constructing the final cfFrag model**

**Table S3 CNV features ranked by their importance**

**Table S4 FSR features ranked by their importance**

**Table S5 FSD features ranked by their importance**

**Table S6 Evaluating the fragmentomics model performances at 85% sensitivity cut-off**
