# Supplementary material for: Cell-free DNA Fragmentomics Assay to Discriminate the Malignancy of Breast Nodules and Evaluate Treatment Response
Source: Genomics Proteomics Bioinformatics. 2025 Apr 4;23(2):qzaf028. doi: 10.1093/gpbjnl/qzaf028 (PMC12321295; doi:10.1093/gpbjnl/qzaf028)
Supplement: qzaf028_Supplementary_Data [file qzaf028_supplementary_data.zip › Table S6.docx]

# Table S6 Evaluating the fragmentomics model performances at 85% sensitivity cut-off

| **Training cohort**  **(5-fold cross-validation)** | | **Actual** | | **Prospective**  **validation cohort** | | **Actual** | |
| --- | --- | --- | --- | --- | --- | --- | --- |
|  |  | **BCs** | **BNs** |  |  | **BCs** | **BNs** |
| **Predict** | **BCs** | 77 | 35 | **Predict** | **BCs** | 118 | 26 |
|  | **BNs** | 14 | 67 |  | **BNs** | 25 | 40 |
| Sensitivity (95% CI) | | 84.6% (75.5%–91.3%) | | Sensitivity (95% CI) | | 82.5% (75.3%–88.4%) | |
| Specificity (95% CI) | | 65.7% (55.6%–74.8%) | | Specificity (95% CI) | | 60.6% (47.8%–72.4%) | |
| PPV (95% CI) | | 68.8% (59.3%–77.2%) | | PPV (95% CI) | | 81.9% (74.7%–87.9%) | |
| NPV (95% CI) | | 82.7% (72.7%–90.2%) | | NPV (95% CI) | | 61.5% (48.6%–73.3%) | |
| Accuracy (95% CI) | | 74.6% (67.9%–80.6%) | | Accuracy (95% CI) | | 75.6% (69.2%–81.3%) | |

*Note*: BC, breast cancer; BN, benign nodule.
