# Supplementary figures and images for: Cell-free DNA Fragmentomics Assay to Discriminate the Malignancy of Breast Nodules and Evaluate Treatment Response
Source: Genomics Proteomics Bioinformatics. 2025 Apr 4;23(2):qzaf028. doi: 10.1093/gpbjnl/qzaf028 (PMC12321295; doi:10.1093/gpbjnl/qzaf028)

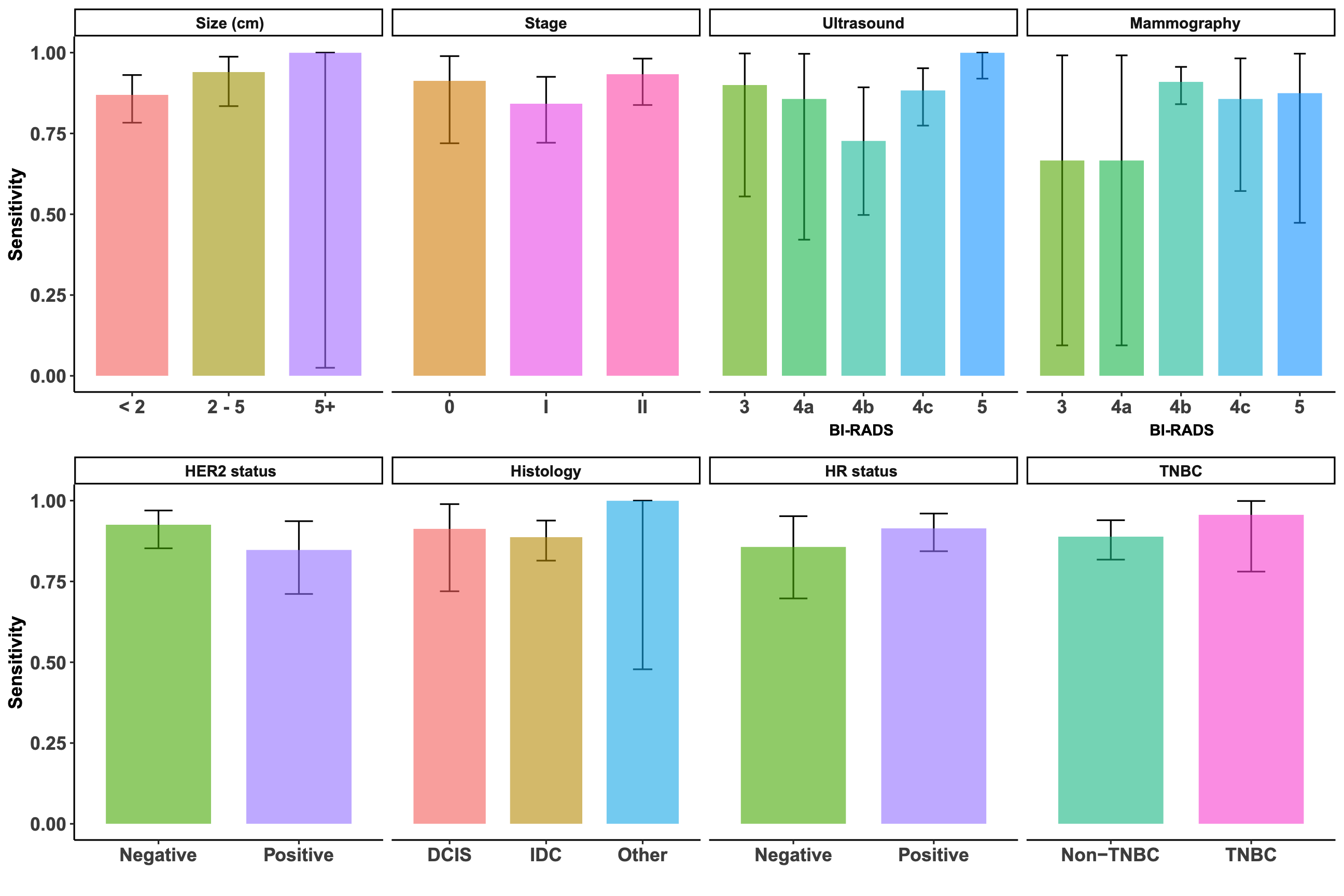

Supplement: qzaf028_Supplementary_Data [file qzaf028_supplementary_data.zip › Figure S10-proof.tiff]

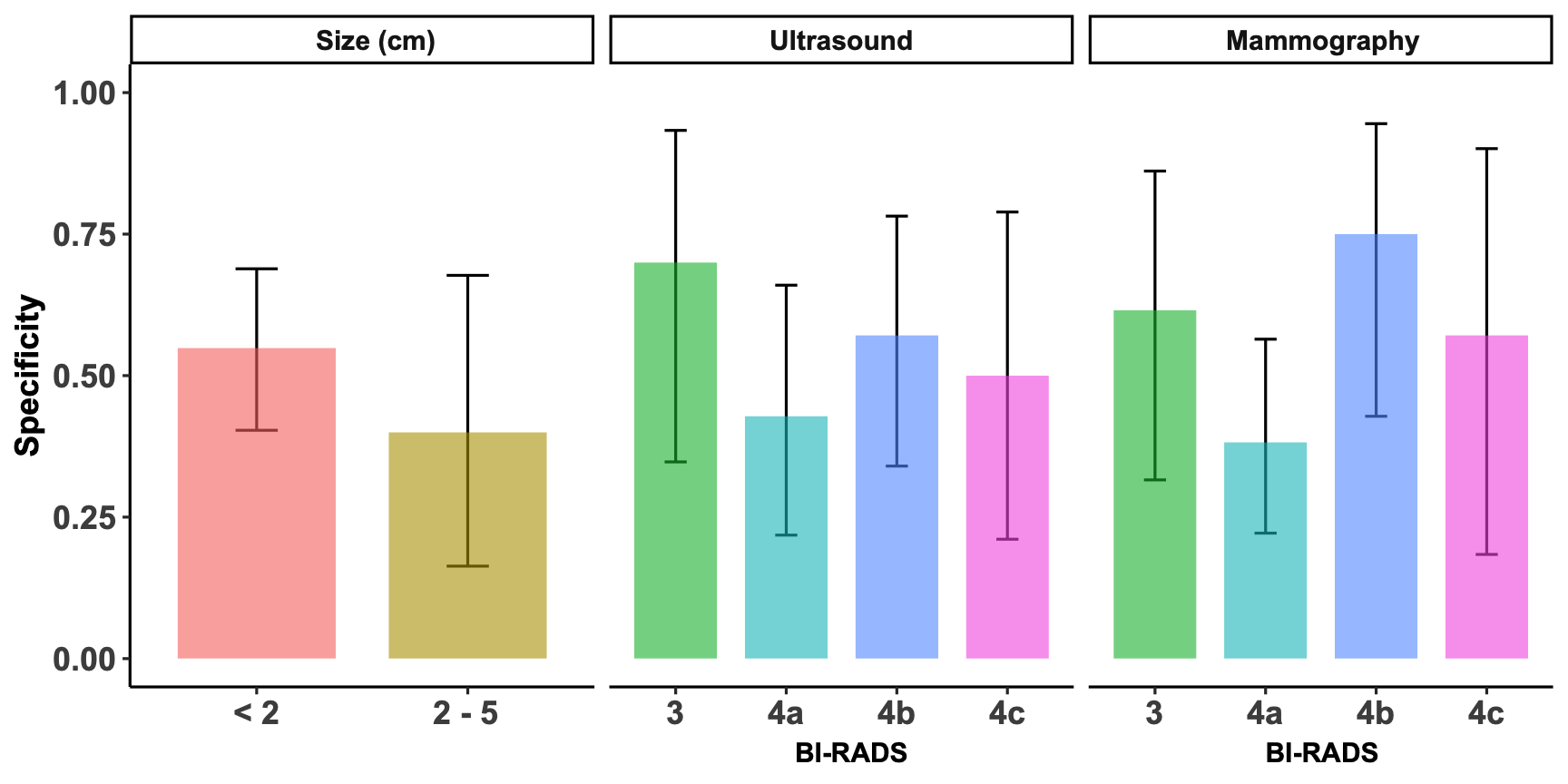

Supplement: qzaf028_Supplementary_Data [file qzaf028_supplementary_data.zip › Figure S11-proof.tiff]

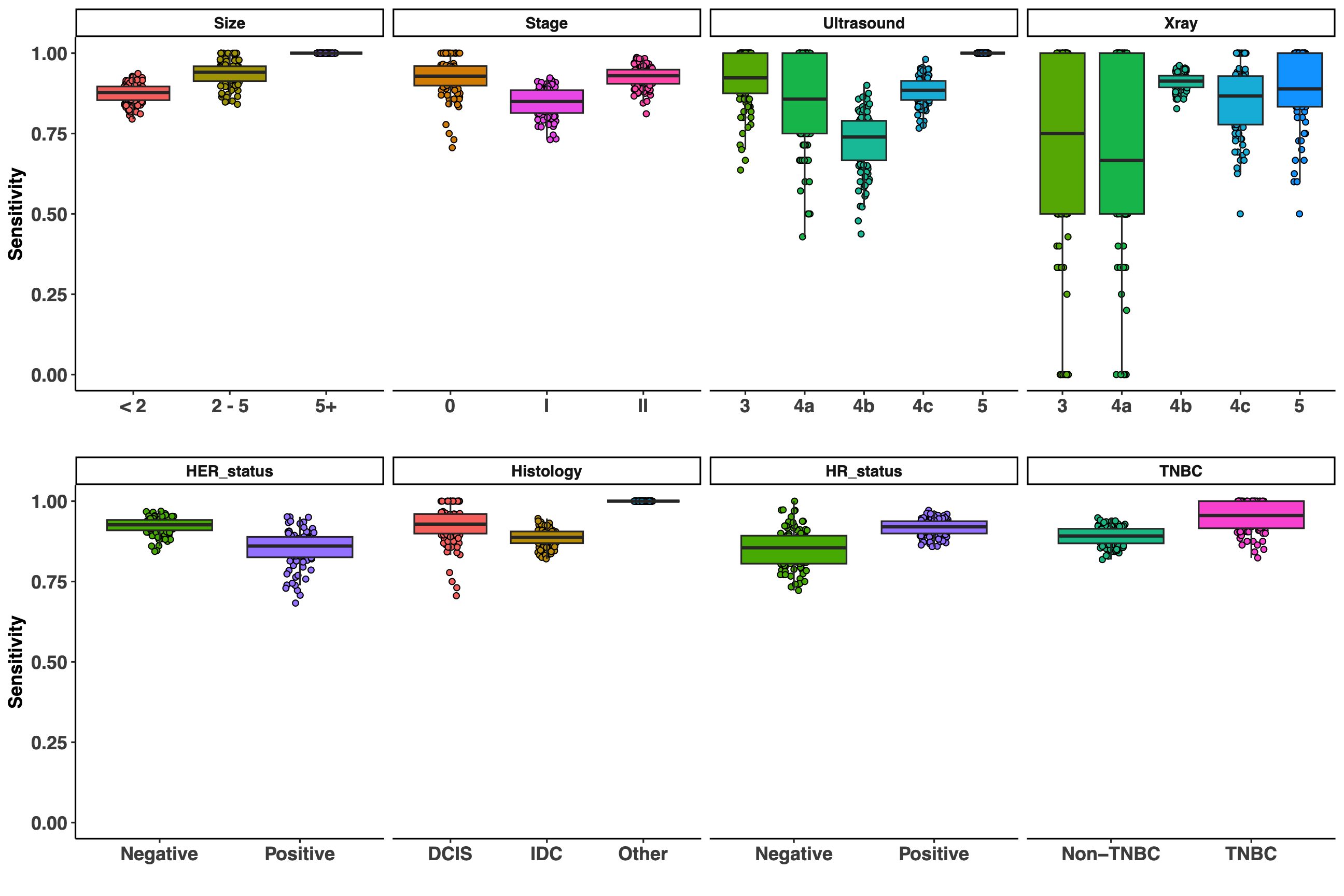

Supplement: qzaf028_Supplementary_Data [file qzaf028_supplementary_data.zip › Figure S12-proof.tiff]

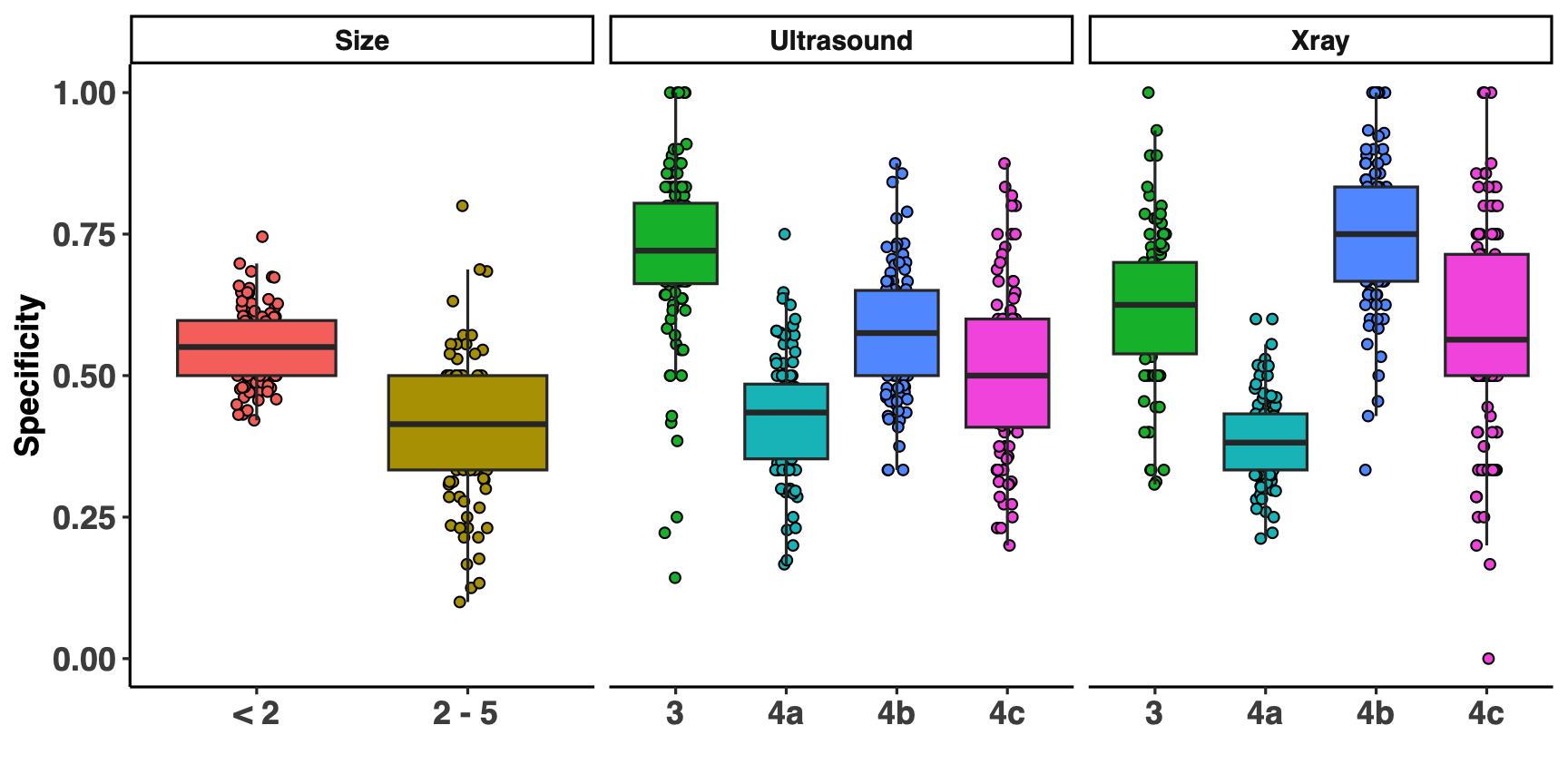

Supplement: qzaf028_Supplementary_Data [file qzaf028_supplementary_data.zip › Figure S13-proof.tiff]

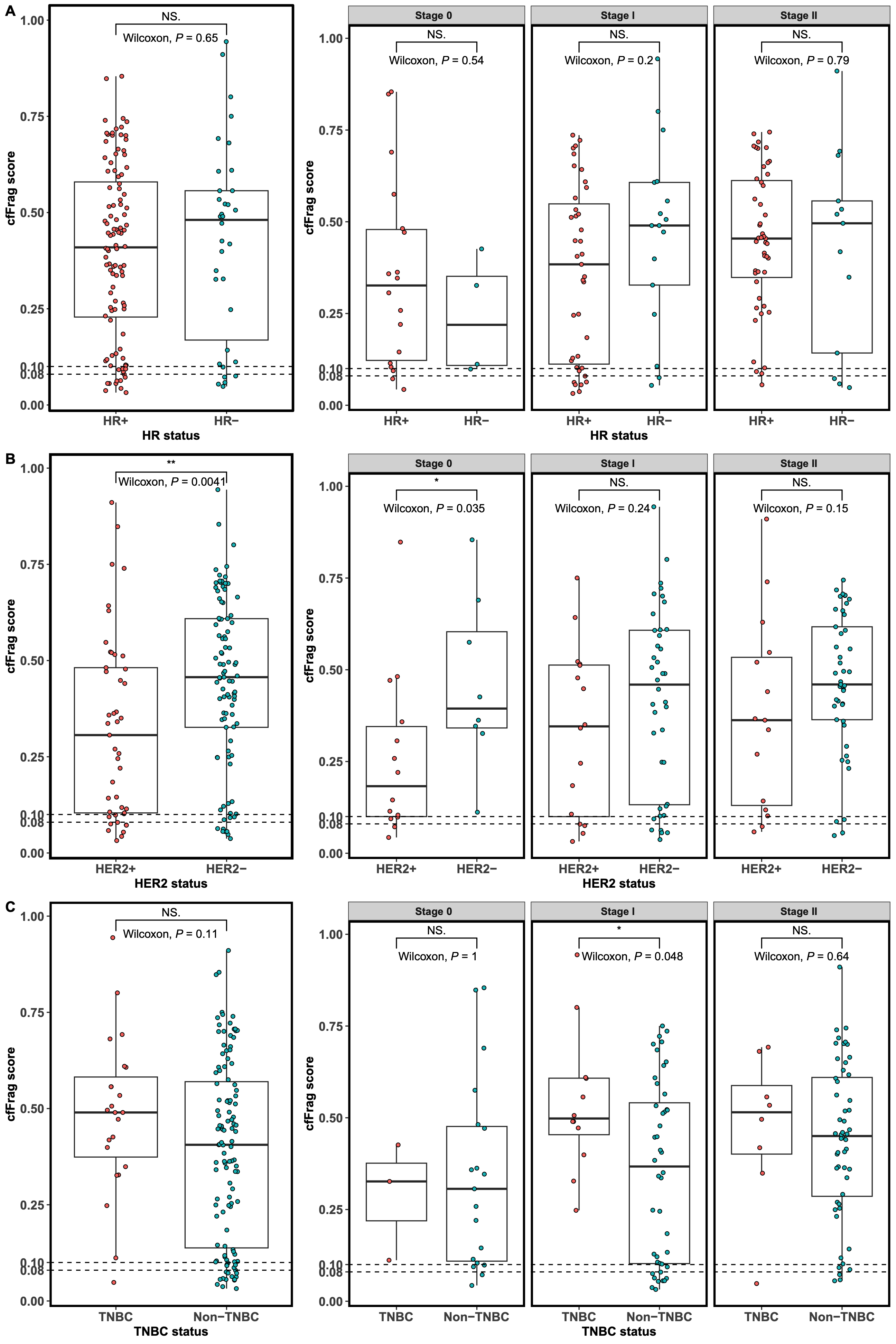

Supplement: qzaf028_Supplementary_Data [file qzaf028_supplementary_data.zip › Figure S14-proof.tiff]

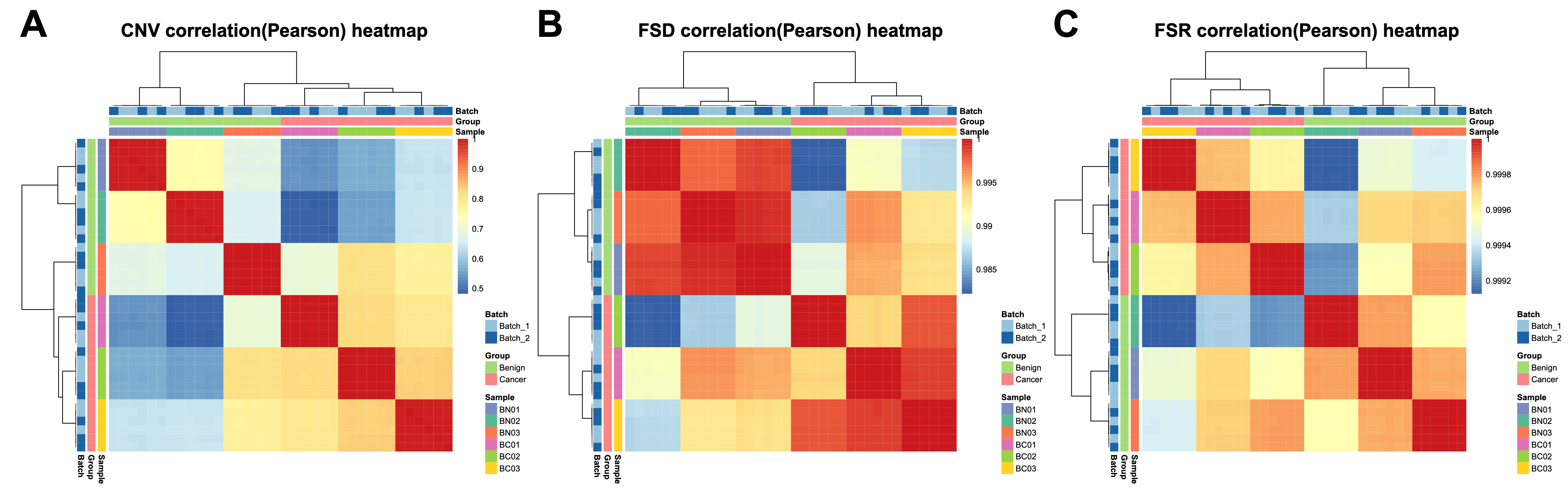

Supplement: qzaf028_Supplementary_Data [file qzaf028_supplementary_data.zip › Figure S15-proof.tiff]

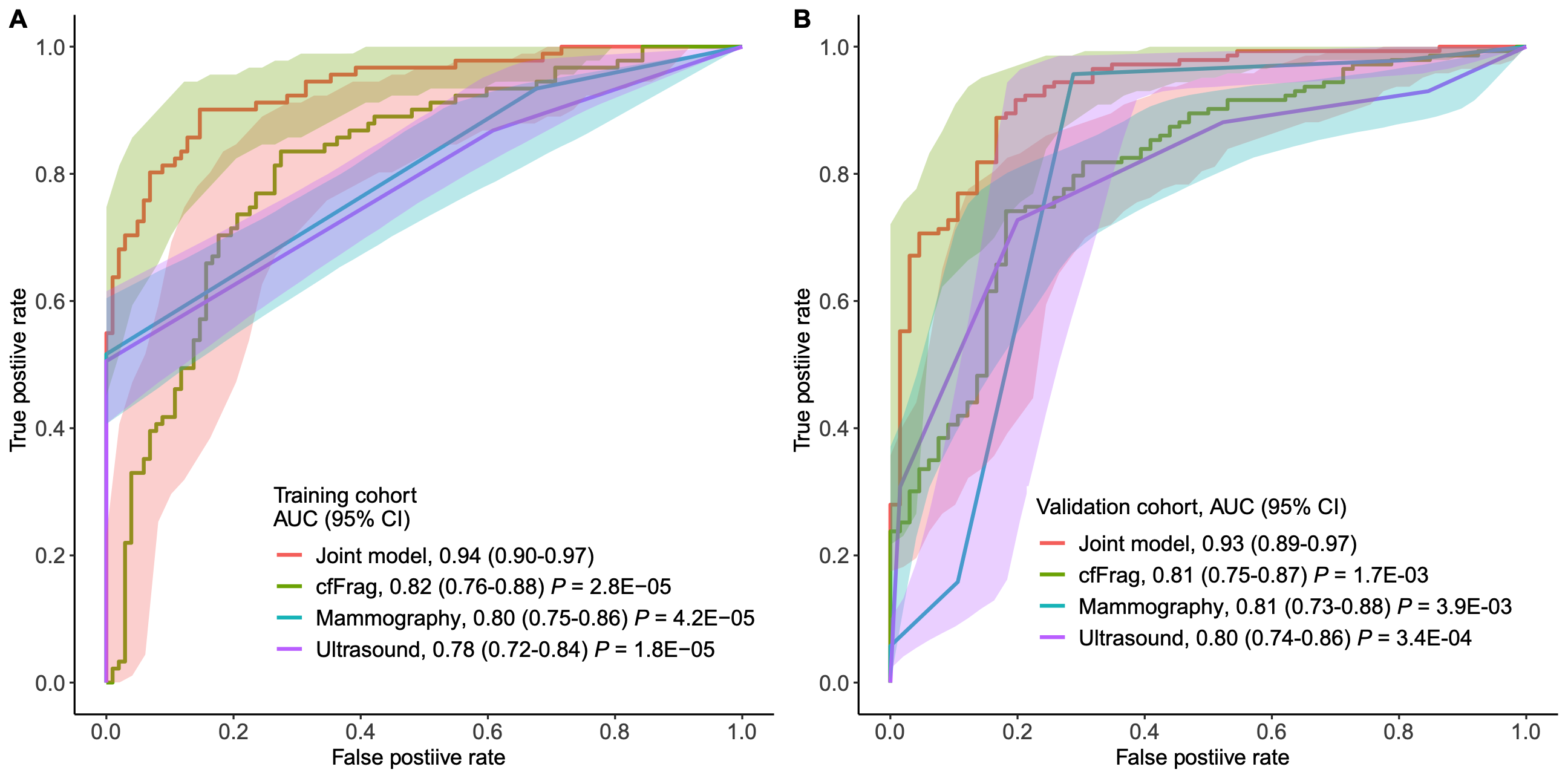

Supplement: qzaf028_Supplementary_Data [file qzaf028_supplementary_data.zip › Figure S16-proof.tiff]

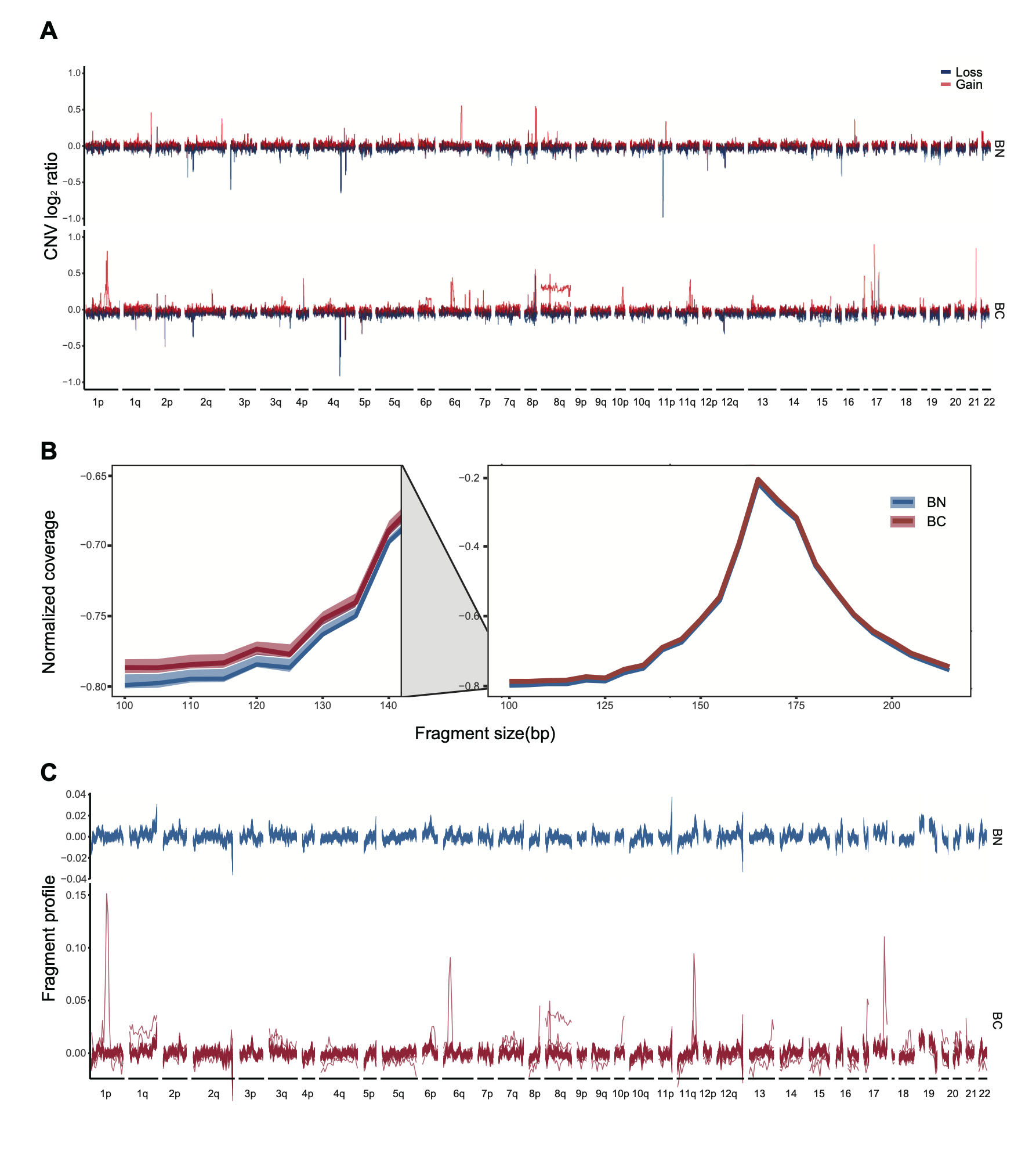

Supplement: qzaf028_Supplementary_Data [file qzaf028_supplementary_data.zip › Figure S1-proof.tiff]

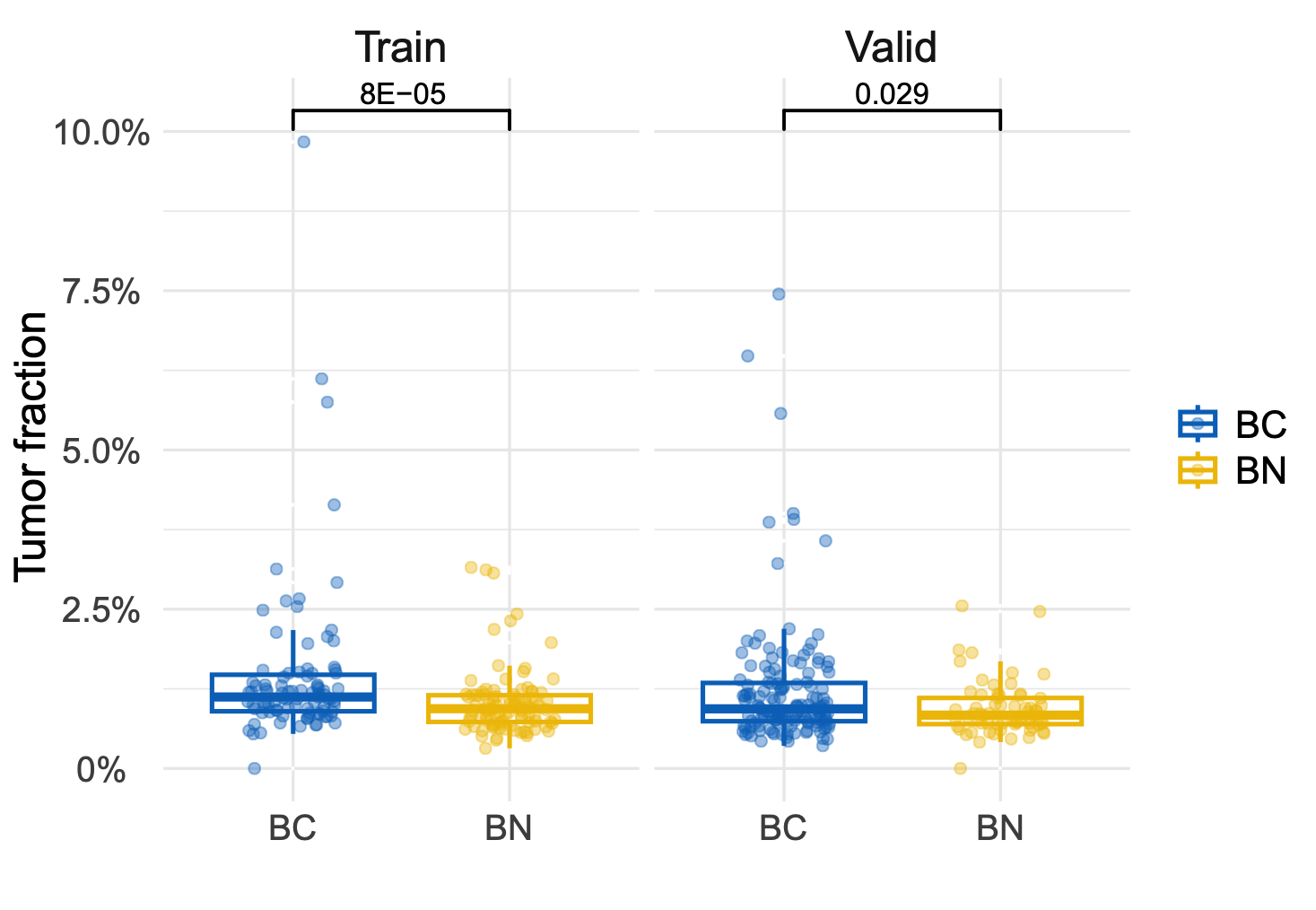

Supplement: qzaf028_Supplementary_Data [file qzaf028_supplementary_data.zip › Figure S2-proof.tiff]

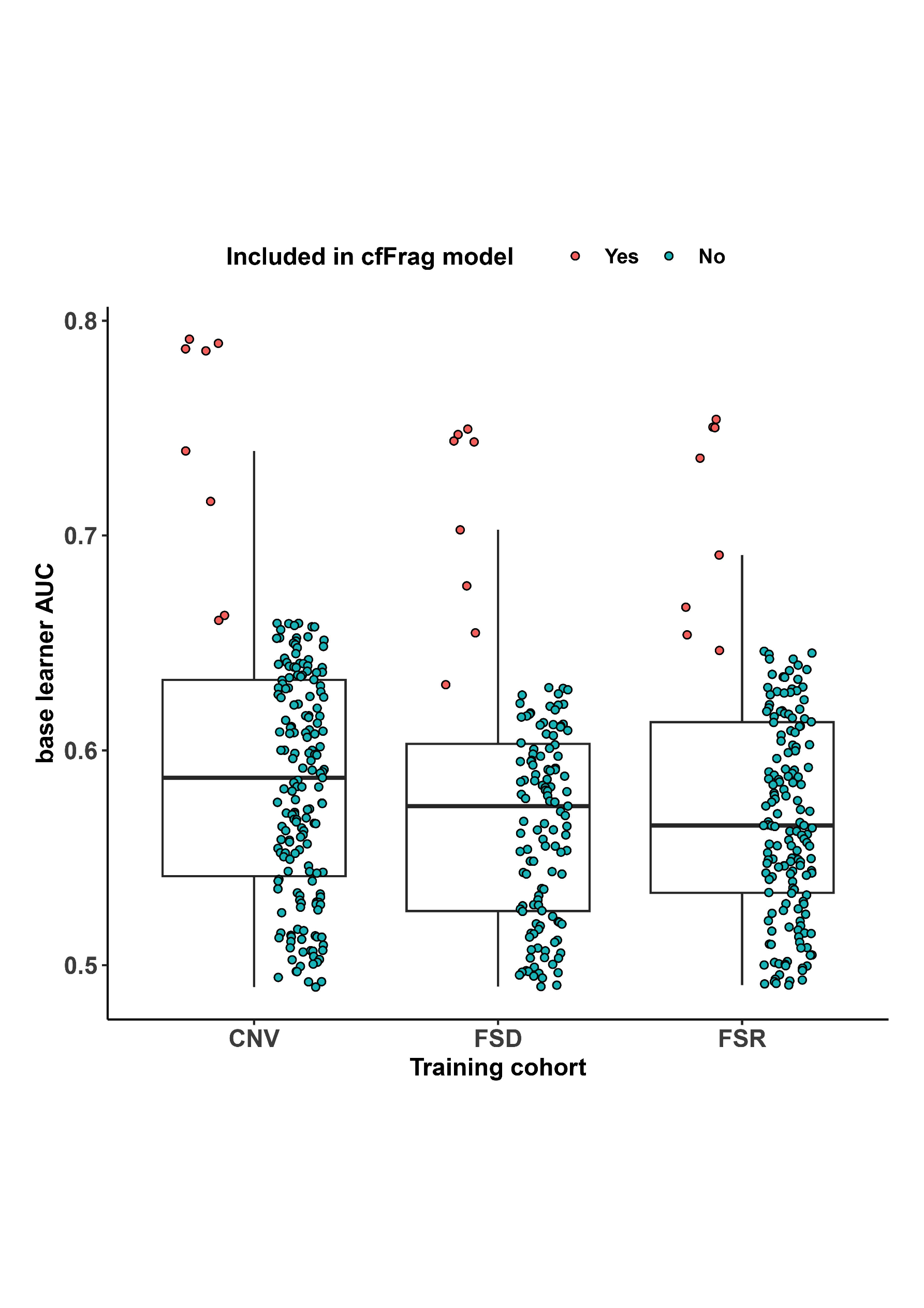

Supplement: qzaf028_Supplementary_Data [file qzaf028_supplementary_data.zip › Figure S3-proof.tiff]

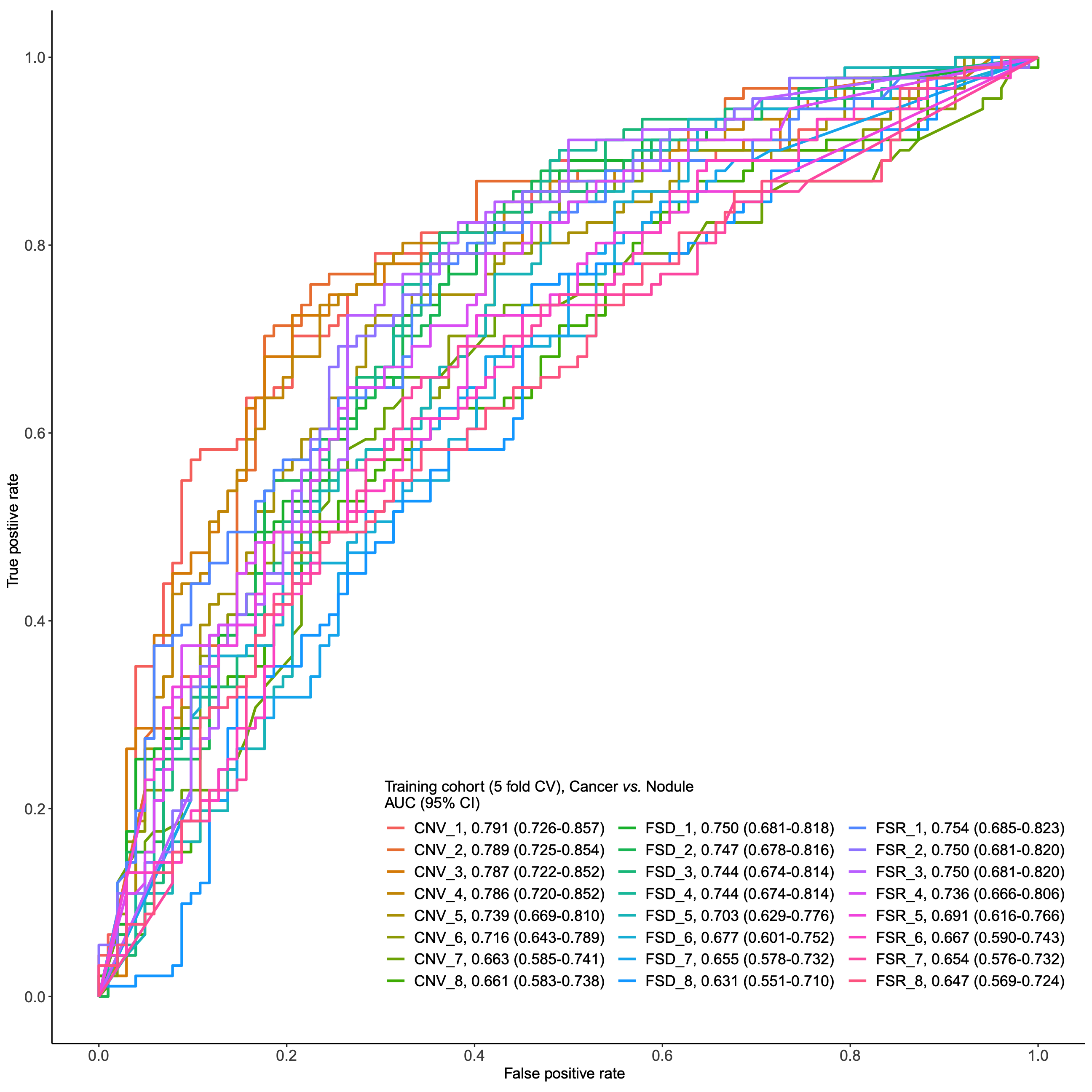

Supplement: qzaf028_Supplementary_Data [file qzaf028_supplementary_data.zip › Figure S4-proof.tiff]

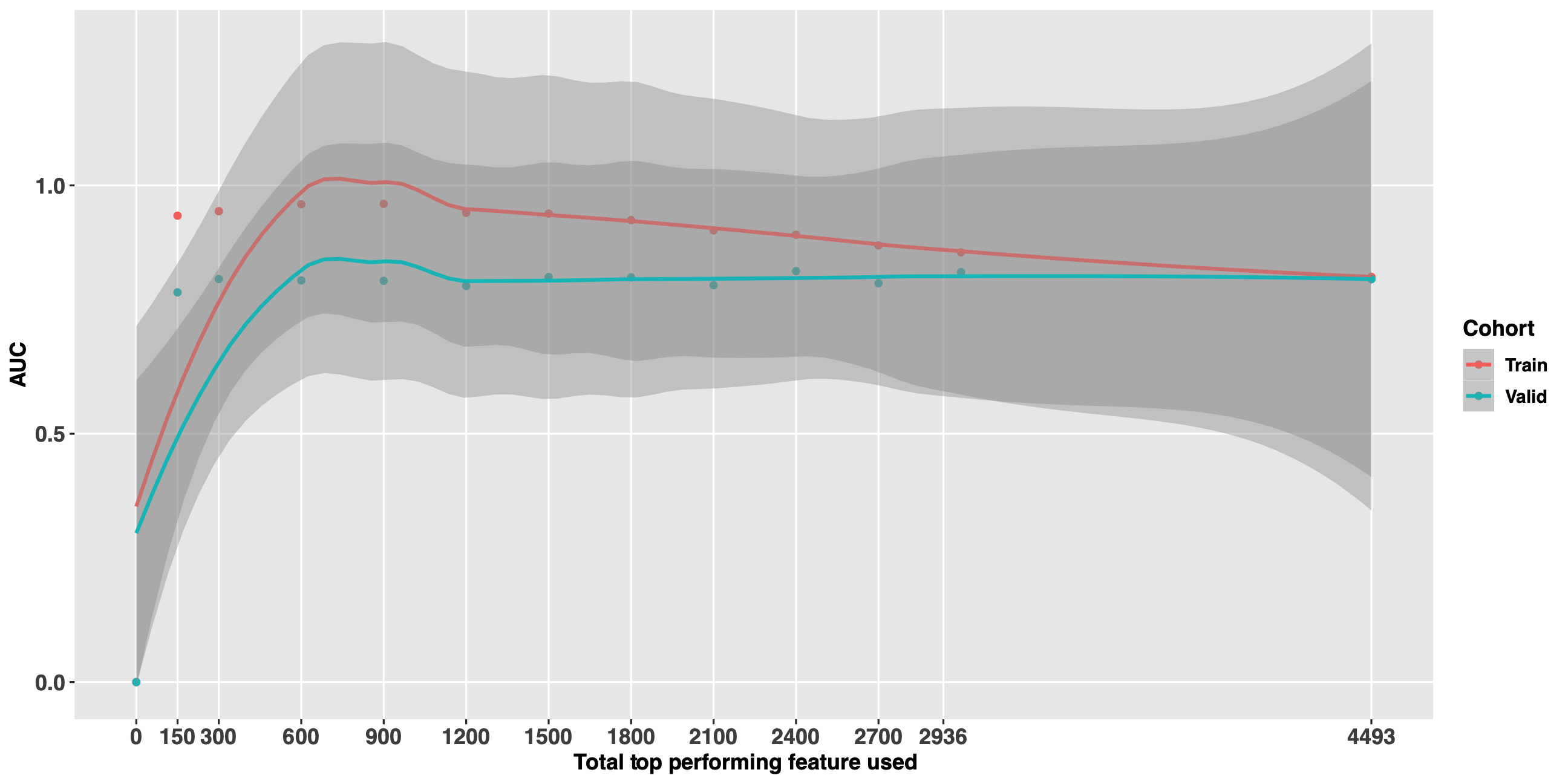

Supplement: qzaf028_Supplementary_Data [file qzaf028_supplementary_data.zip › Figure S5-proof.tiff]

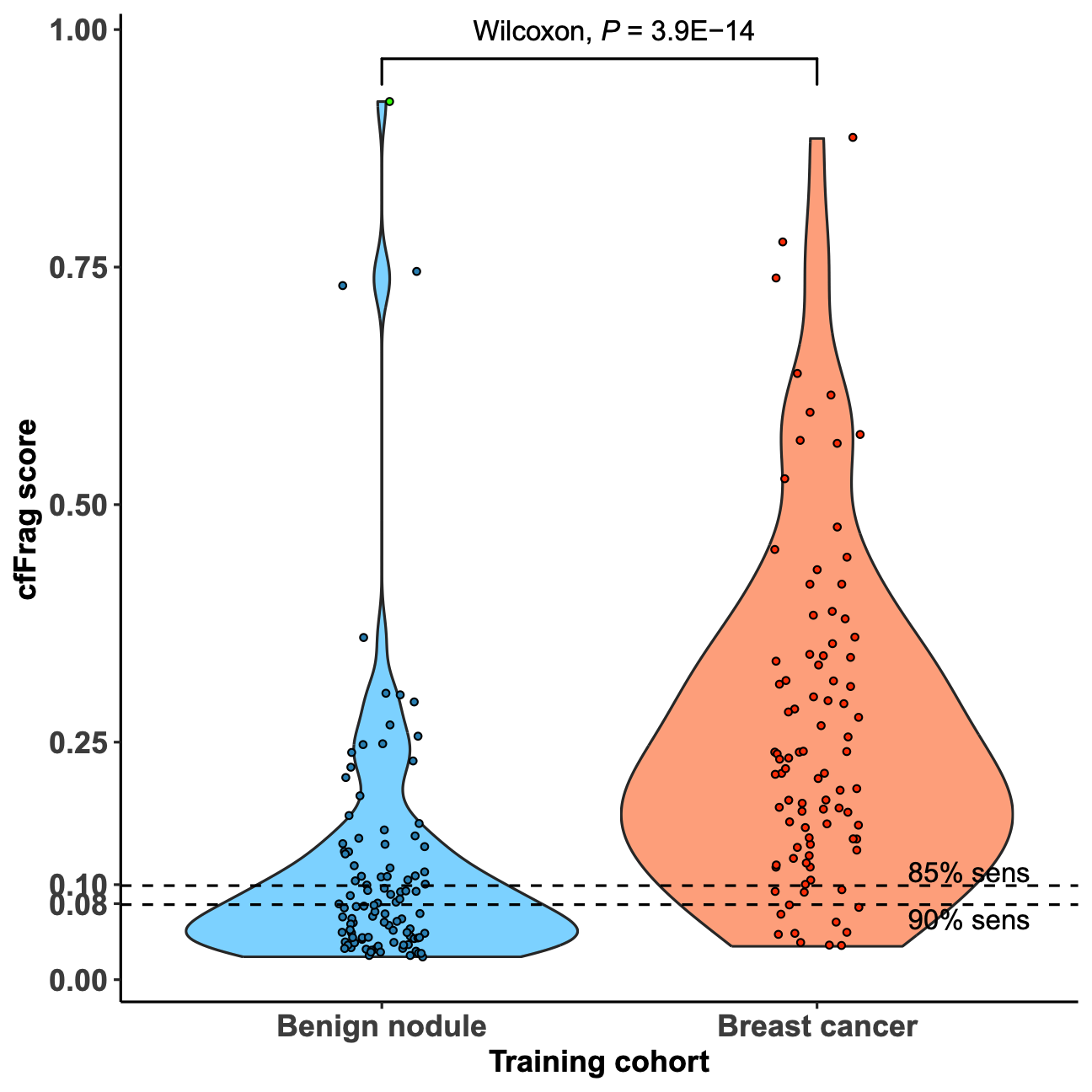

Supplement: qzaf028_Supplementary_Data [file qzaf028_supplementary_data.zip › Figure S6-proof.tiff]

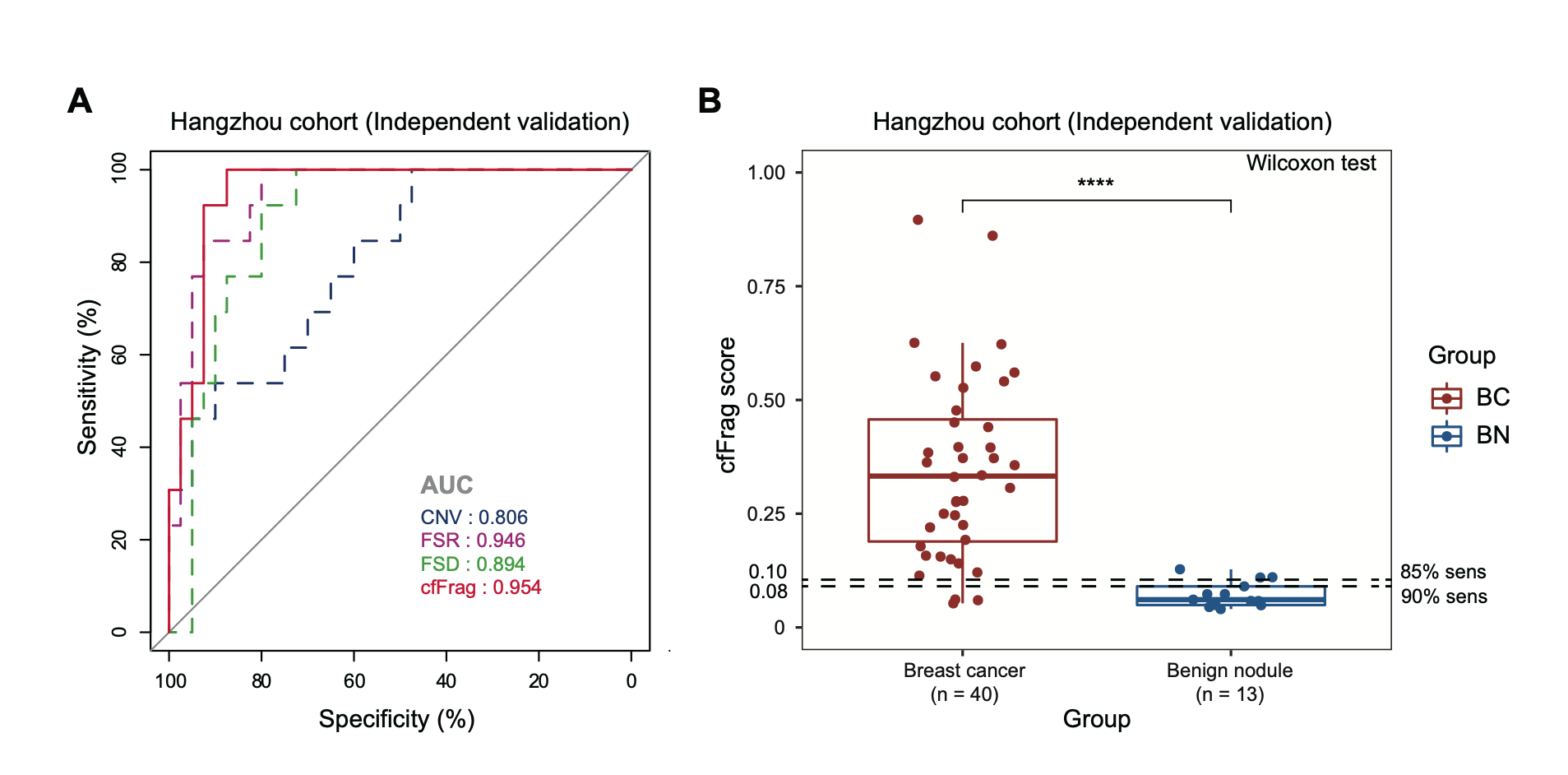

Supplement: qzaf028_Supplementary_Data [file qzaf028_supplementary_data.zip › Figure S7-proof.tiff]

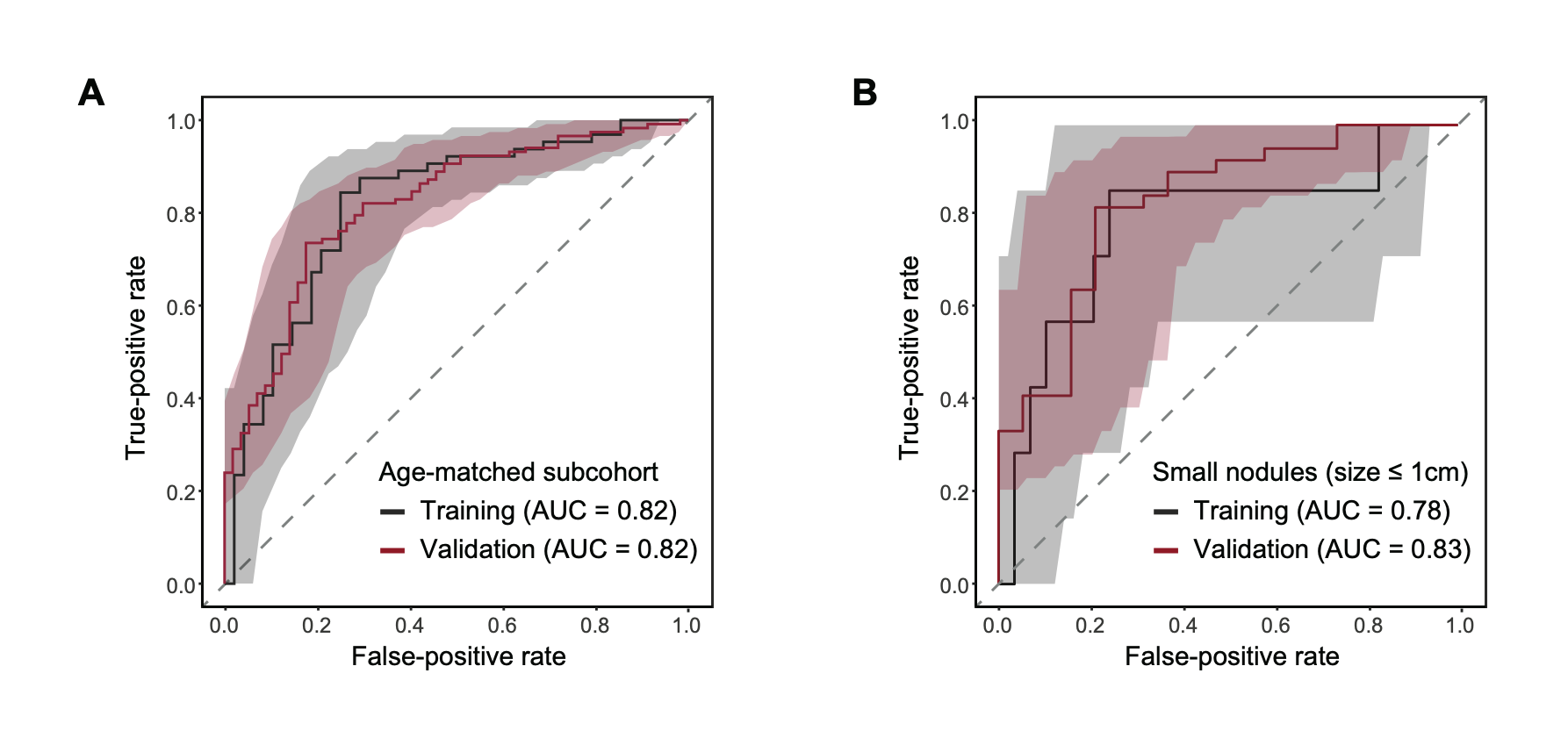

Supplement: qzaf028_Supplementary_Data [file qzaf028_supplementary_data.zip › Figure S8-proof.tiff]

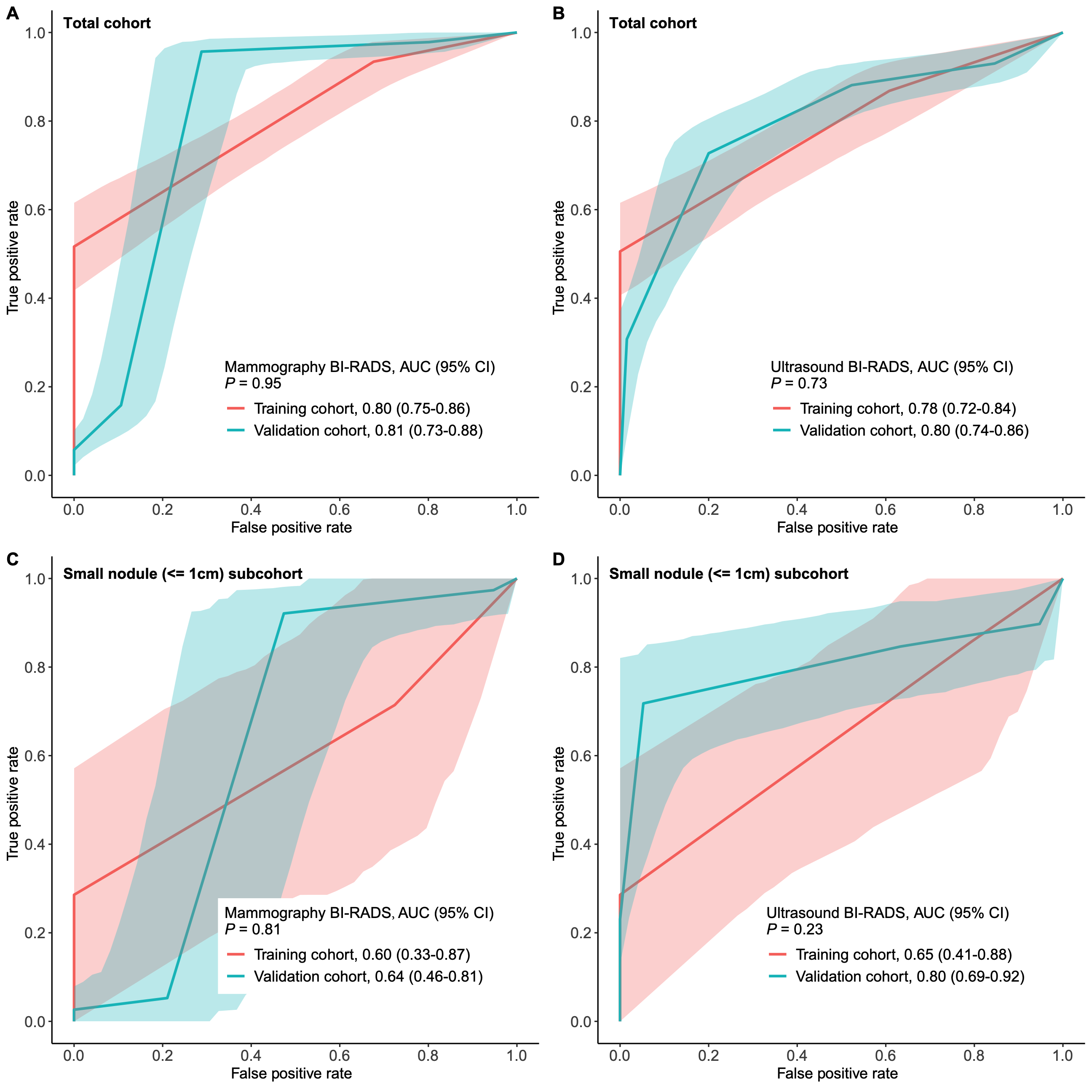

Supplement: qzaf028_Supplementary_Data [file qzaf028_supplementary_data.zip › Figure S9-proof.tiff]
